# Steroid Glycosides Hyrcanoside and Deglucohyrcanoside: On Isolation, Structural Identification, and Anticancer Activity

**DOI:** 10.3390/foods10010136

**Published:** 2021-01-11

**Authors:** Silvie Rimpelová, Tomáš Zimmermann, Pavel B. Drašar, Bohumil Dolenský, Jiří Bejček, Eva Kmoníčková, Petra Cihlářová, Soňa Gurská, Lucie Kuklíková, Marián Hajdůch, Tomáš Ruml, Lubomír Opletal, Petr Džubák, Michal Jurášek

**Affiliations:** 1 Department Biochemistry and Microbiology, University of Chemistry and Technology Prague, Technická 5, 166 28 Prague, Czech Republic; silvie.rimpelova@vscht.cz (S.R.); jiri.bejcek@vscht.cz (J.B.); lucie.kuklikova@vscht.cz (L.K.); tomas.ruml@vscht.cz (T.R.); 2 Department of Chemistry of Natural Compounds, University of Chemistry and Technology Prague, Technická 5, 166 28 Prague, Czech Republic; tomas.zimmermann@vscht.cz (T.Z.); pavel.drasar@vscht.cz (P.B.D.); Petra.Cihlarova@vscht.cz (P.C.); 3 Department of Analytical Chemistry, University of Chemistry and Technology Prague, Technická 5, 166 28 Prague, Czech Republic; bohumil.dolensky@vscht.cz; 4 Department of Pharmacology and Toxicology, Faculty of Medicine in Pilsen, Charles University, Alej Svobody 76, 323 00 Pilsen, Czech Republic; eva.kmonickova@lfp.cuni.cz; 5 Department of Pharmacology, Second Faculty of Medicine, Charles University, Plzeňská 311, 150 00 Prague, Czech Republic; 6 Institute of Molecular and Translational Medicine, Faculty of Medicine and Dentistry, Palacký University and University Hospital in Olomouc, Hněvotínská 976/3, 779 00 Olomouc, Czech Republic; sona.gurska@upol.cz (S.G.); marian.hajduch@upol.cz (M.H.); 7 Department of Pharmaceutical Botany, Charles University, Akademika Heyrovského 1203, 500 05 Hradec Králové, Czech Republic; opletal@faf.cuni.cz

**Keywords:** cardiac glycosides, secondary plant metabolites, natural product isolation, hyrcanoside, deglucohyrcanoside, ouabain, cymarin, digitoxin, anticancer activity, Na^+^/K^+^-ATPase inhibitors

## Abstract

Cardiac glycosides (CGs) represent a group of sundry compounds of natural origin. Most CGs are potent inhibitors of Na^+^/K^+^-ATPase, and some are routinely utilized in the treatment of various cardiac conditions. Biological activities of other lesser known CGs have not been fully explored yet. Interestingly, the anticancer potential of some CGs was revealed and thereby, some of these compounds are now being evaluated for drug repositioning. However, high systemic toxicity and low cancer cell selectivity of the clinically used CGs have severely limited their utilization in cancer treatment so far. Therefore, in this study, we have focused on two poorly described CGs: hyrcanoside and deglucohyrcanoside. We elaborated on their isolation, structural identification, and cytotoxicity evaluation in a panel of cancerous and noncancerous cell lines, and on their potential to induce cell cycle arrest in the G2/M phase. The activity of hyrcanoside and deglucohyrcanoside was compared to three other CGs: ouabain, digitoxin, and cymarin. Furthermore, by in silico modeling, interaction of these CGs with Na^+^/K^+^-ATPase was also studied. Hopefully, these compounds could serve not only as a research tool for Na^+^/K^+^-ATPase inhibition, but also as novel cancer therapeutics.

## 1. Introduction

Cancer is responsible for many people’s deaths each year, and it represents the second most common cause of human death worldwide [1]. Even though this disease’s incidence and mortality have declined over the past 20 years, there is still no reliable therapy for eradicating it. Recently, in addition to the traditional drug discovery approach, a novel strategy called drug repositioning has emerged. It lies in using the old for a novel purpose. This approach is economically much more feasible and faster than the traditional approach of drug approval [2].

Interestingly, drug repositioning has also been used for some cardiac glycosides (CGs) as a potential remedy for cancer. Several undergoing clinical trials on CG administration for cancer in mono or combination therapy can be found in [3]. Moreover, also promising is the fact that a lower incidence in some types of cancer in patients on CG therapy (cardiac conditions) has been reported [4]. While another study claims the opposite [5], others stand somewhere in between [6,7]. However, what is quite certain and makes CGs a new hope for cancer treatment are clinical data showing that CGs, mainly digoxin, significantly prolong the survival of cancer patients otherwise treated with traditional chemotherapeutics. What is also beneficial is the fact that CGs at multiple levels affect the immune response and trigger immunogenic cell death, which significantly contributes to their anticancer activity [8].

CGs are secondary plant metabolites found mainly in *Digitalis purpurea* L. and *Digitalis lanata* Ehrh. (digitoxin, Dg; digoxin), *Strophanthus gratus* (ouabain, Ob), and *Nerium oleander* L. (oleandrin). Their biological effect is associated with the interaction with Na^+^/K^+^-ATPase (NKA), the integral membrane protein of animal cells maintaining the balance of sodium and potassium ions. The CG pharmacophore is the structure of 5β,14β-androstane-3β,14-diol, which is substituted at the C-3 position by a saccharide moiety and the C-17 position by an unsaturated lactone [9]. According to the type of lactone, CGs are classified into cardenolides and bufadienolides; 5β,14β-androstane-3β,14-diol may be substituted in some other positions [10]. Biological activity has been shown to decrease after saturation of the lactone double bond [11,12]. Furthermore, biological activity is also affected by the type and number of carbohydrate units at the C-3 position, and it increases with the decreasing number of carbohydrate units [13,14]. The only exception is aglycone, the biological activity of which is lower than that of its glycosylated variants [13,15].

As aforementioned, CGs, as a unique group of metabolites, have been extensively utilized for the treatment of various heart conditions, and recently, they were also explored as possible anticancer agents (reviewed in [16]). Based on the positive ionotropic effect they induce, they are introduced as drugs in the treatment of heart failure and cardiac arrhythmias. The ionotropic effect is associated with NKA inhibition, which leads to an increase in intracellular Na^+^ concentration, resulting in an augmented influx of Ca^2+^ ions into the cell, followed by contraction of the heart muscle [17]. However, the interaction of CGs with NKA may not only be associated with disruption of Ca^2+^ homeostasis, as SERCA (Sarco-Endoplasmic Reticulum Calcium ATPase) inhibitors do [18]. It has also been found that at low (subclinical) concentrations of CGs, where there is little or no inhibition of NKA, this enzyme can serve as a receptor that activates non-receptor tyrosine kinases. This in turn leads to activation of mitogen-activated protein kinase (MAPK) and the triggering of the Ras/MAPK signaling pathway [19,20]. This further leads to stimulation or inhibition of cell proliferation, depending on the cell type: cancer cell proliferation is inhibited, while primary noncancerous cells are not [21,22,23].

Besides this, CGs are also potent activators of the immune system response by induction of immunogenic cell death, which is a tremendous advantage over some other currently used chemotherapeutics, such as cisplatin, which lacks this effect. The immunogenic cell death is achieved by calreticulin exposure to the cell surface and the secretion of ATP and high-mobility group box 1 protein [24].

One of the widely studied CGs in terms of cancer is digoxin, currently included in a clinical trial for cancer combination therapy, in which it is co-administered with cisplatin (ClinicalTrials.gov). Another interesting and, in cancer treatment, possibly potent CG is Dg. This cardenolide type of CG also binds and inhibits NKA [25], and was shown to be potent in inhibiting cancer cell proliferation and cell cycle arrest [26,27,28] already at low nanomolar concentrations. Such concentrations are commonly found in the blood plasma of patients treated with Dg due to heart failure [13,14,15].

**Figure 1:**
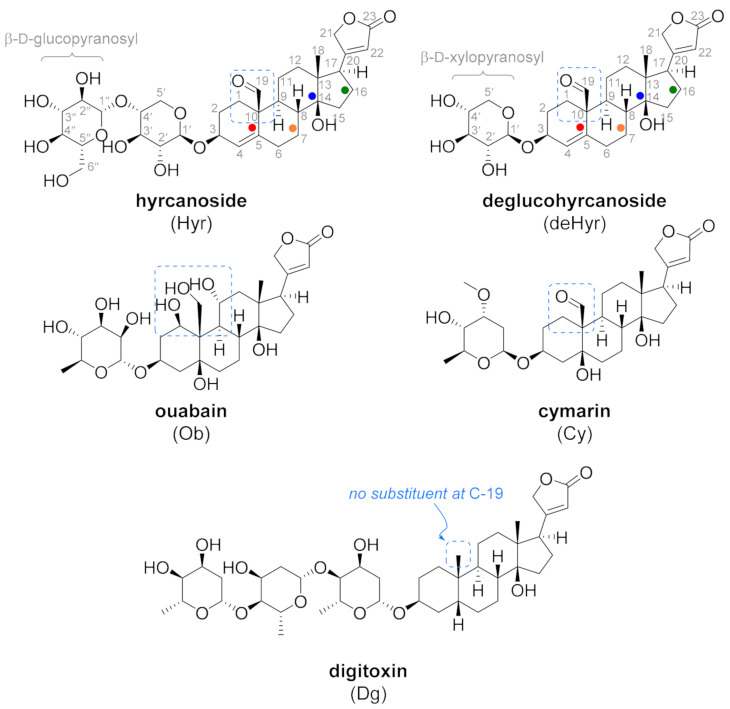
Structures of natural C-19-oxo cardiotonics and digitoxin. Carbon numbering of Hyr and deHyr is in grey. The ring designation of the steroid ring is highlighted by dots (i.e., the A ring is marked by a 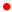 dot, B with 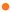, C with 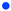, and D with 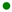 dot).

However, both Dg and digoxin lack cancer cell selectivity, resulting in high systemic toxicity often encountered in patients on CG therapy. Based on this, the seeking of novel CGs with improved properties and enhanced performance has not yet been finished. Therefore, we report on two very interesting CGs: hyrcanoside (Hyr, Figure 1) and deglucohyrcanoside (deHyr, Figure 1), about which there has not yet been much information. Hyr is a secondary plant metabolite of *Coronilla varia* L. Regarding cytotoxicity, the only information available is on Hyr-containing alcoholic extracts inhibiting the growth of KB cell [29] and human lymphocytic leukemia (P-388) and nasopharynx carcinomas (9KB) [30]. From earlier reports, it is obvious that deglucohyrcanoside acts as digoxin, but its cytotoxicity is several times lower [31,32,33], which indicates the potential of this CG as a therapeutic used in cancer treatment.

## 2. Materials and Methods

### 2.1. Materials

For thin-layer chromatography (TLC), aluminum silica gel sheets for detection in UV light (TLC Silica gel 60 F254, Merck, Prague, Czech Republic) were used. For TLC visualization, a diluted solution of H_2_SO_4_ in MeOH was used, and plates were heated. For column chromatography, 30–60 μm silica gel (ICN Biomedicals, Costa Mesa, CA, USA) was used. The NMR spectra were recorded by a 500 MHz instrument (JEOL, Tokyo, Japan) at 25 °C. The chemical shifts (*δ*) are presented in ppm, and the coupling constants (*J*) are presented in Hz. The ^1^H and ^13^C chemical shifts are referenced to tetramethylsilane using the solvent signals CHD_2_SOCD_3_ 2.50 ppm, CD_3_SOCD_3_ 39.52 ppm, CHD_2_OD 3.31 ppm, and CD_3_OD 49.00 ppm. For the signal assignments and obtaining of the coupling constants, combinations of the following standard NMR sequences were used: 1D and selective homodecoupled ^1^H NMR spectra, ^13^C NMR spectra with or without ^1^H BB decoupling, 2D gCOSY, gTOCSY, dqf-COSY with 25% non-uniform sampling (NUS), 2D NOESY and ROESY (mix time 400 and 350 ms), 2D gHSQC and gHMBC (7 Hz) with adiabatic pulses and 25% NUS, 1D selective gNOESY1D and gROESY1D (various mix time), and gTOCSY1D (10, 15, 20, 40, 80, 160 ms). The content of Hyr and deHyr (dried extracts were dissolved in 50% MeOH) was verified using mass spectrometry, employing direct injection into the electrospray ionization source of QTRAP 6500+ (AB Sciex, Framingham, MA, USA) with a Turbo V Ion source. The mass spectrometer was operated in a positive scan mode with *m*/*z* ranging between 100 and 1000. The ion source settings were as follows: a temperature of 200 °C, a capillary voltage of 5500 V, curtain gas of 20 psi, nebulizer gas, and heater gas of 30 psi. The data were acquired and evaluated using Analyst 1.6.3 software (AB Sciex, Framingham, MA, USA). In further LC-MS analyses, the Quadrupole LC/MS (ESI ionization) with the Infinity III LC system (Agilent Technologies, Santa Clara, CA, USA) was used for LR-MS and HPLC analyses (C18 column: 100 mm, and UV detection).

### 2.2. Extraction of C. varia Seeds and Isolation of Compounds

Seeds of crown vetch (*Coronilla varia* L., Fabaceae, sometimes also *Securigera varia* (L.) Lassen, or *Coronilla pendula* Kit.) purchased from Agrostis Trávníky Ltd., Rousínov u Vyškova (CZ) were ground on an electric blade coffee grinder to a fine powder. Powdered seeds (500 g) were mixed with ethanol (500 mL) and kept at room temperature for seven days. Then, the mixture was filtered through frita, the filtrate was evaporated, re-dissolved in 200 mL of EtOH, and transferred onto a chromatography silicagel column (length of 24 cm, a diameter of 6 cm, 800 g of silica gel). Seed contents were eluted at first by ethanol and then by a gradient mixture of EtOH-H_2_O (10:1→ 3:1, *v*/*v*), respectively. Thus, two fractions were obtained: ethanolic one (H1) and fractions eluted by the mixture of aqueous EtOH (H2). MS and TLC evaluations of fraction H1 showed that mainly daphnoretin, scopoletin, and umbelliferone (structures are shown in Figure S1) were present (after evaporation, a dry residue of 85 g) [29]. On the other hand, fraction H2 (we obtained a brown-red syrupy evaporate of 70 g) contained solely traces of the aforementioned three compounds plus (−)-epicatechin (Figure S1), deHyr, and some unidentified polar compounds [29]. As a major component, Hyr was identified. The content of both fractions was subjected to column chromatography.

Chromatographic purification of H1 (84 g) over a silica gel column (length of 24 cm, a diameter of 6 cm, 800 g of silica gel, using DCM-MeOH, 20:1→ 3:2, (*v*/*v*) as eluent) afforded mostly triacylglycerols, as expected, and some minor products. Only about 120 mg of crude Hyr was obtained. This was in accordance with the preliminary LC-MS spectra recorded for fraction H1, which showed only a negligible amount of compounds of interest. On the contrary, chromatographic purification of 69 g of the second major fraction H2 (length of 30 cm, a diameter of 4 cm, 600 g of silica gel, using DCM-MeOH, 10:1→ 3:2, *v/v* as an eluent) provided four fractions (f1–f4), and LC-MS confirmed the presence of both deHyr (in fraction 2) and Hyr (in fraction 4).

In addition, other known compounds contained in *C. varia* were detected by LC-MS in fraction f2, namely daphnoretin and umbelliferone in fraction f1 and (−)-epicatechin and scopoletin in f3 (structures are shown in Figure S1). DeHyr and Hyr fractions were further purified. Hyr fraction f4 of H2 was purified using a silica gel column (length of 30 cm, a diameter of 4 cm, 600 g of silicagel, using DCM-MeOH, 10:1→ 6:1, (*v*/*v*) as eluent) to provide fairly pure Hyr. Due to the fact that Hyr contains both hydrophilic disaccharide and lipophilic steroidal moiety, standard chromatographic purification was not efficient enough to provide pure Hyr (or deHyr). Thus, the final processing step was the crystallization of Hyr from the solution. Finally, the crude in absolute MeOH was dissolved, and the careful addition of Et_2_O showed signs of crystals being formed. Crude Hyr was thus dissolved in MeOH (100 mL), then, Et_2_O (50 mL) was slowly added and the solution left to stay at 4 °C overnight. The white solids formed were fritted and washed with ether. Hyr was obtained as white crystals (1.634 g, 2.40 mmol; 3.3%). Purified deHyr fraction H1 (length of 30 cm, a diameter of 4 cm, 600 g of silica gel, using DCM-MeOH, 10:1, *v*/*v* as an eluent) provided a notably lower amount of crude deHyr. This product was also crystallized by dissolving it in a mixture of MeOH (10 mL) and acetone (20 mL) with the subsequent addition of Et_2_O (20 mL), then it was left overnight at 4 °C. This process provided deHyr as white crystals (96 mg, 0.19 mmol; 0.2%). MS analyses confirmed the corresponding molecular composition (Figures S6 and S7). The purity and identity of the isolated compounds were checked by LC analysis (Figures S8 and S9) and NMR (Section 3.1) spectroscopy, respectively.

### 2.3. In Silico Modeling

The Maestro program 2019-3 (Schrödinger, LLC, New York, NY, USA) was used for all structural modifications and subsequent molecular docking of the tested CGs into NKA. The CG structures were obtained from the ChemSpider database (www.chemspider.com). Using the LigPrep module, the missing hydrogen atoms were added to the ligands. Then, the ligands’ structure was converted to 3D, and their energy was minimized using the OPLS3e force field. The structure of NKA with the code name 4RET (organism: *Sus scrofa*) was obtained from the ProteinDataBank database (www.rcsb.org). Non-protein moieties (aspartyl phosphate, cholesterol, sucrose, digoxin, Mg^2+^, *N*-acetyl-d-glucosamine, phospholipids, and water) were removed from the NKA structure. This was followed by adding hydrogen atoms to the NKA structure. Then, the amino acids were assigned a protonation state corresponding to pH = 7 using the PROPKA function, and the energy of the molecule was minimized by the OPLS3e force field. Using the amino acids l-Thr_114_, l-Asp_121_, and l-Thr_797_, a ligand-binding site was defined, which consisted of two cubes with an edge length of 15 and 35 Å (a small and large cube, respectively). The center of the molecule should not leave the smaller cube and the molecule as a whole should not leave the larger cube. Subsequently, a subset of a spherical pocket with a radius of 4 Å was defined using the constraints function, into which the C and D cycles of the steroid skeleton were fixed. The CG ligands were docked into NKA with an extra precision mode.

### 2.4. Cell Lines

If not indicated otherwise, the cell lines were purchased from the American Type Culture Collection (ATCC). The CCRF-CEM line is derived from T-cell childhood acute lymphoblastic leukemia, which shows the highest chemosensitivity in our tumor cell lines panel. K562 is the erythroid-myeloid precursor cell line derived from chronic myeloid leukemia carrying the *BCR-ABL* hybrid gene. A549, MCF-7, PC-3, 5637, U-2 OS, and MiaPaCa-2 are cells derived from lung, breast, prostate, bladder carcinoma, osteosarcoma, and pancreatic adenocarcinoma, respectively. HEK 293T are transformed human embryonic kidney cells, and L929 (Sigma, St. Louis, MS, USA) are transformed mouse fibroblasts. HCT116, cells from colorectal carcinoma, and their *p53* gene knock-out variant (HCT116p53-/-) were purchased from Horizon Discovery. This cell line is a model of human tumors bearing *p53* loss-of-function mutations or biallelic deletion of the *p53* gene, frequently associated with poor prognosis. The MRC-5 and BJ cells were used as noncancerous cells; more specifically, they are human fibroblasts from the lungs and foreskin, respectively. According to the supplier’s recommendations, all cells were cultured at 37 °C in a 5% CO_2_ atmosphere and 100% humidity. The culture media used were: DMEM, RPMI 1640, and MEM (according to a cell line); all were supplemented with 5 g L^−1^ glucose, 10% fetal calf serum, 2 mM glutamine, 100 U mL^−1^ penicillin, 100 µg mL^−1^ streptomycin, and NaHCO_3_. Cells were passaged every two or three days using 0.25% trypsin plus 0.01% EDTA (ethylenediamine tetraacetic acid) in phosphate-buffered saline.

### 2.5. MTS Cytotoxic Assay

To evaluate compound cytotoxicity, cells were seeded in 384-well microtiter plates in a volume of 30 mL. The next day, aliquots of the tested derivatives were transferred with Echo550 acoustic liquid handler (Labcyte) to obtain dose response curves with dilution factor 4. The experiments were performed in technical duplicates and three or more biological replicates. After 72 h of incubation in a humidified incubator, 4 mL of the MTS/PMS stock solution were pipetted into each well. After another 1–4 h of incubation, the absorbance at 490 nm was measured using an EnVision Multilabel Plate Reader (PerkinElmer). IC_50_ values were calculated from the appropriate dose response curves in Dotmatics software using the following equation: IC_50_ = (OD_drugexposed well_/mean ODc_ontrol wells_) × 100% [34].

### 2.6. Mice and Peritoneal Primary Cells

Female mice of the inbred strain C57BL/6, eight to ten weeks old, were purchased from Charles River Deutschland (Sulzfeld, Germany). They were kept in transparent plastic cages in groups of ten. The animals were housed with food and water ad libitum, lighting was set on 6–18 h, and the temperature was set at 22 °C. All protocols were approved by the institutional ethics committee (MSMT15894/2013-310). Animals, killed by cervical dislocation, were intraperitoneally injected with 8 mL of sterile saline. Pooled peritoneal cells collected from mice were washed in sterile saline, re-suspended in the culture medium, and seeded into 96-well microplates in final 100-μL volumes (Costar, Cambridge, MA, USA). The final density of the cells was 0.25 × 10^6^ per mL^−1^. The cultures were maintained with or without compounds for 24 h at 37 °C with 5% CO_2_ in a humidified Heraeus incubator in complete RPMI-1640 (Merck-Sigma, St. Louis, MS, USA), which contained 10% heat-inactivated fetal bovine serum, 2 mM of l-glutamine, 50 μg·mL^−1^ of gentamicin, and 5 × 10^−5^ M of 2-mercaptoethanol (all Merck-Sigma, St. Louis, MS, USA). Compounds (Ob, Cy (cymarin), Hyr, deHyr) were prepared as 100 mM stock solutions in dimethyl sulfoxide (DMSO) with cell culture grade. The next dilution continued immediately before the experiment with the culture medium. To eliminate the influence of DMSO, equal levels of DMSO were added to the experimental groups.

### 2.7. Viability of Mouse Primary Cells

The viability of mouse peritoneal cells was determined using a colorimetric assay based on the cleavage of the tetrazolium salt WST-1 by mitochondrial dehydrogenases in viable cells (Merck-Sigma, St. Louis, MS, USA). The cells were cultured as described above. After 24 h of culture, the WST-1 was added, and the cells were kept in the Heraeus incubator at 37 °C for an additional 3 h. Optical density at 450/690 nm was determined. The cytotoxicity of the tested CGs was expressed as a percentage. The test samples were related to control samples consisting of untreated cells and samples with 100% dead cells evoked by 1% Triton, according to the formula: ((exp. value − control value)/(Triton value − control value)) × 100. All control and experimental variants were run in quadruplicates. The data were analyzed using GraphPad Prism software 6.05 (GraphPad, San Diego, CA, USA). Values were expressed as the mean ± standard error of the mean (SEM).

### 2.8. Analysis of Cell Cycle Arrest and Cell Death

CCRF-CEM cells were seeded at a density of 1 × 10^6^ cells per one mL in six-well plates (TPP) and treated the next day with CGs at concentrations corresponding to 1× or 5× the IC_50_ value. Together with the CG-treated cells, a vehicle-treated sample was harvested at the same time point. After 24 h, the cells were washed with cold phosphate-buffered saline, fixed dropwise in 70% ethanol, and stored overnight at −20 °C. The cells were then washed with hypotonic citrate buffer, treated with RNAse (50 µg mL^−1^), and stained with propidium iodide. Flow cytometry using a 488 nm laser (FACS-Calibur, Becton Dickinson, NJ, USA) was used for measurement. The cell cycle was analyzed by the ModFitLT program (Verity), and apoptosis was measured in a logarithmic model expressing the percentage of particles with a lower propidium content than cells in the G0/G1 phase (<G1) of the cell cycle in the CellQuest program (Becton Dickinson). Half of the sample was used to label cells with pH3^Ser10^-FITC antibody (Exbio) for subsequent flow cytometry analysis of mitotic cells [35].

### 2.9. BrDU Incorporation Analysis

The cells were cultured in the same way as the cell cycle analysis method. Just before harvesting, 5-bromo-2′-deoxyuridine (BrDU) of 10 µM concentration was added to the cells for pulse-labeling for 30 min. Then, the cells were fixed with −20 °C cold 70% ethanol and stored in a freezer for 16 h. Before antibody staining, the samples were incubated on ice for 30 min, washed once with phosphate-buffered saline (PBS), and re-suspended in 2 M of HCl for 30 min at room temperature to hydrolyze their DNA. After neutralization with 0.1 M of Na_2_B_4_O_7_ (borax) solution, the cells were washed with PBS containing 0.5% Tween-20 and 1% BSA. This was followed by staining with the primary anti-BrDU antibody (Exbio) for 30 min at room temperature. Then, the cells were washed with PBS and stained with a secondary anti-mouse antibody conjugated to fluorescein isothiocyanate (Merck-Sigma, St. Louis, MS, USA) at room temperature in the dark. After another washing with PBS and incubation with propidium iodide (0.1 mg·mL^−1^) and RNAse A (0.5 mg·mL^−1^) for 1 h at room temperature in the dark, the cells were analyzed by flow cytometry using a 488 nm laser (FACSCalibur, Becton Dickinson, Franklin Lakes, NJ, USA) [35].

### 2.10. BrU Incorporation Analysis

The cells were cultured and processed as described above. Before harvesting, the cells were pulse-labeled with 1 mM of 5-bromouridine (BrU) for 30 min. The cells were then fixed in 1% buffered paraformaldehyde with 0.05% NP-40 at room temperature for 15 min, and then stored at 4 °C overnight. Before measurement, they were washed with 1% glycine in PBS, washed again with PBS, and stained with primary anti-BrdU antibody cross-reactive to BrU (Exbio) for 30 min at room temperature in the dark. From this point on, the experiment was performed exactly as in the method described above [35].

## 3. Results and Discussion

### 3.1. Isolation and Identification of Hyr and deHyr

**Figure 2:**
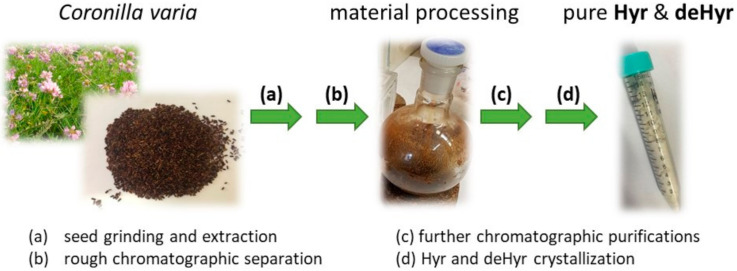
Diagram presenting the process leading to the isolation of Hyr and deHyr from the seeds of *Coronilla varia*.

Isolation of the desired substances from the plant seeds consisted of several steps (Figure 2). First, it was necessary to grind the seeds and extract them. This procedure is described in detail in Section 2.1. Two extraction solvent systems were used: EtOH and aqueous EtOH, providing two extracts designated as H1 and H2, respectively. According to MS and TLC analyses, the desired substances (Hyr and deHyr) were mainly present in the H2 fraction. This was followed by purification of the extracts by chromatographic separation on silica gel. Separation of the components from extract H2 yielded pure Hyr (3.3%) and a very small amount of deHyr (0.0002%). Hembree et al. [30] described the isolation of Hyr and deHyr from 14.8 kg of powdered seeds with the yield of semi-pure Hyr of 2.57 g (0.00017%) and 70 mg (0.000005%) of deHyr. We hypothesize that the low yield of deHyr can be explained by its absence in the plant material, and the trace amount is formed during the processing. Taken together, our procedure has improved the yield of pure Hyr and deHyr by ca. 20,000 and 40 times, respectively.

A thorough literature search has revealed that the name hyrcanoside has also been used for other chemical structures. Two different compounds were named hyrcanoside (Figure S2). A cardenolide, (3β)-3-[(4-*O*-β-d-glucopyranosyl-β-d-xylopyranosyl)oxy]-14-hydroxy-19-oxocarda-4,20(22)-dienolide (Hyr) (CAS Registry No. 15001-93-1; Figure 2 and  Figure S2—compound Hyr), mostly named hyrcanoside (*Coronilla*) [30,31], which is the substance isolated from *Coronilla varia* (or from *C. varia* Prilipko, a synonym of *Securigera cretica* (L.) Lassen, *Securigera securidaca* (L.) Degen et Dörfler, and some other plants) [36], and a phenolglycoside that was isolated from *Dorema hyrcanum* Koso-Pol. or *Dorema glabrum* Fisch. & C.A. Mey., 1-[2-[(6-*O*-α-d-glucopyranosyl-β-d-glucopyranosyl)oxy]-6-hydroxy-4-methoxy-phenyl]ethanone (CAS Registry No. 60197-59-3; Figure S2—compound s1) [37,38]. Hereby, we suggest naming this compound hyrcanoside (*Dorema*).

To make things even more complicated, Abubakirov [39] lists the structure of Hyr (Figure 1 and  Figure S2) named as securidaside, and declares it identical with steroidal hyrcanoside. However, Zatula et al., in a series of earlier research articles [40,41,42], presents securidaside as C4(5) saturated, 5α,11β-hydroxy derivative s2 (CAS Registry No. 18309-58-5; for structure, see Figure S2). Zatula et al. in 1969 corrected [43] the structure to be identical with Hyr, from which it was cited by Abubakirov [39].

We also attempted to confirm the molecular structures of Hyr and deHyr by ^1^H and ^13^C NMR spectra (Table S1). Unfortunately, our values of the ^13^C chemical shifts were not in full accordance with the ones recorded in DMSO-*d*_6_ [30], nor in CD_3_OD [44] (Hyr is named as securigenin glycoside s3; see Figures S4 and S5). Thus, we performed the signal assignment of all ^13^C and ^1^H signals of both Hyr and deHyr in both solvents. By the combination of 2D HSQC, HMBC, NOESY, TOCSY, and dqf-COSY spectra, followed by the series of the selective 1D TOCSY, NOESY, ROESY, and homodecoupled ^1^H spectra, we were able to assign all of the signals, including stereo positions (Table S1). We concluded that our assignments and characteristics of Hyr are fully consistent with its known crystal structure and molecular models [44]. Comparison of ^1^H and ^13^C chemical shifts of Hyr in CD_3_OD revealed that the chemical shifts of C3ʹ and H3ʹ signals are interchanged with C5ʹʹ and H5ʹʹ, respectively [44]. After this correction, the ^13^C chemical shifts differed from −0.15 to 0.03 ppm, and ^1^H chemical shifts differed from −0.01 to −0.08 ppm; thus, we concluded that our Hyr isolated from *C. varia* and the securigenin glycoside s3 (= Hyr, Figure S3; the designation is different for data comparison) [44] isolated from *S. securidaca* are identical compounds. In contrast, the values of ^13^C chemical shifts of Hyr differed more significantly in DMSO-*d*_6_ [30]. Even when the obvious interchange of the signals for C4 and C22 were corrected, there were still several deviations (1–11 ppm), which cannot be removed by an interchange. Since the compound was isolated from the same herb and its structure was confirmed by comparison with the synthetic standard, we concluded that the ^13^C chemical shifts are confused in [30].

### 3.2. In Silico Modeling

**Figure 3:**
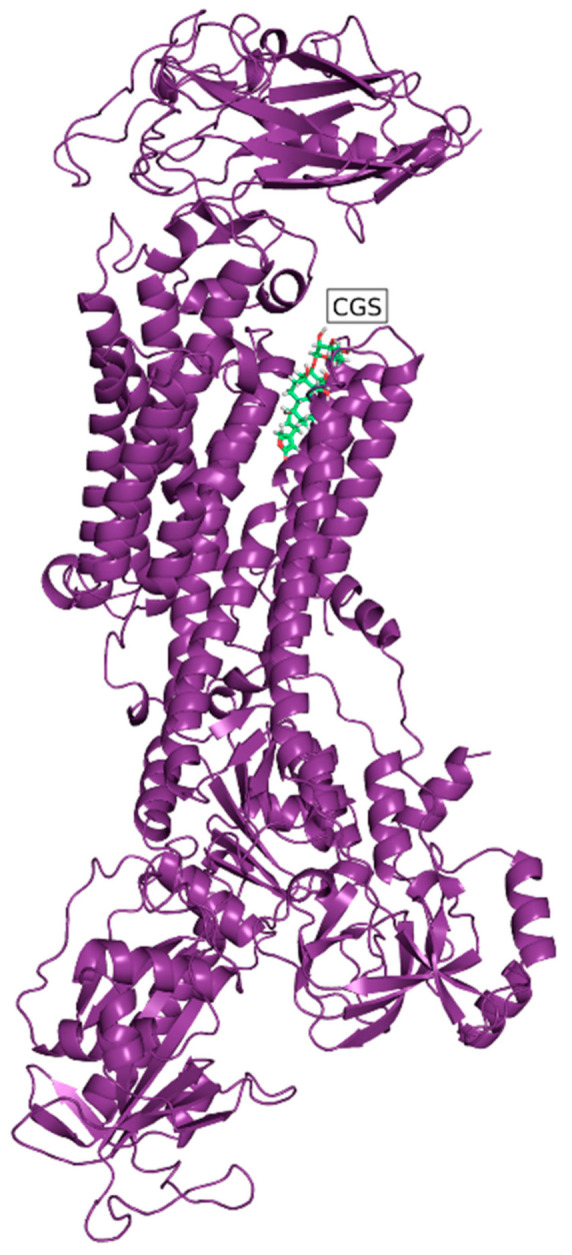
Far view of ouabain (green) docked into Na^+^/K^+^-ATPase (purple) with the lowest binding energy. Images were taken using PyMOL 2.3.3 (Schrödinger, LLC, New York, NY, USA).

**Figure 4:**
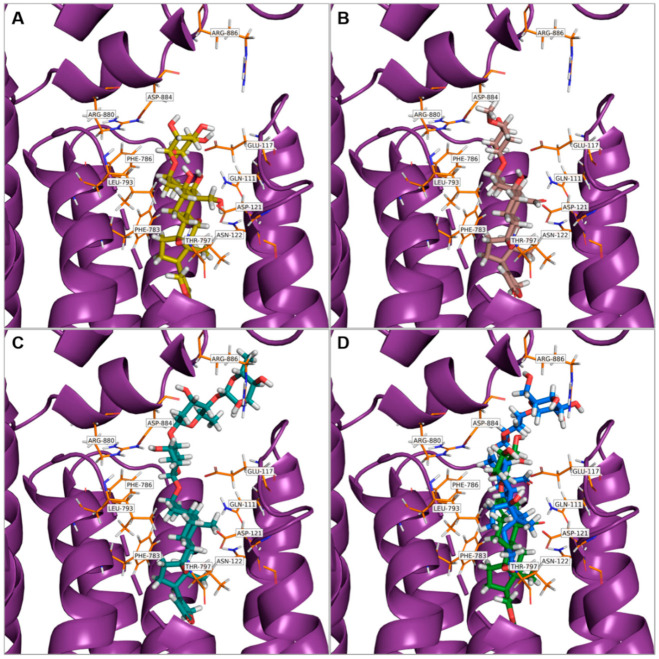
Near view of cardiac glycosides (stick representations) docked into Na^+^/K^+^-ATPase (purple, image representations; orange, individual residues, stick representations) with the lowest binding energy mode. The cardiac glycoside binding site in the Na^+^/K^+^-ATPase is indicated. (**A**) Ouabain, (**B**) cymarin, (**C**) digitoxin, (**D**) hyrcanoside (in blue) and deglucohyrcanoside (in green). The images were taken using PyMOL 2.3.3 (Schrödinger, LLC, New York, NY, USA).

Since CGs are well-known to be NKA inhibitors, we strived to determine whether Hyr and deHyr share the same fate. Using molecular docking, which is a method for studying protein-ligand complex interactions, we performed an in silico study of these CGs and NKA. The data were compared with Ob, Dg, and Cy. The structures of all five examined NKA ligands consisted of a steroid skeleton, which is substituted by a lactone and a saccharide moiety at the C-17 and C-3 position, respectively. Hyr and deHyr were docked into the NKA binding site, which is located in the transmembrane domain of the NKA α-subunit between the helices one to six. This binding site is divided into a polar (l-Gln_111_, l-Glu_117_, l-Asp_121_, l-Asn_122_, and l-Thr_797_) and nonpolar part (l-Ile_315_, l-Phe_316_, l-Gly_319_, l-Phe_783_, l-Phe_786_, and l-Leu_793_) [45]. In Figure 3 and Figure 4, the far and near views of the CGs docked into NKA are shown in the lowest binding energy mode (Table 1). From Table 1, based on the binding energies, it is clear that the NKA-Ob and NKA-Hyr complexes were the most and the least stable ones, respectively, from the docked ligands.

The NKA-Ob complex, which served as a reference, contained a hydrogen bridge between the conserved β-hydroxyl group at the C-14 position and l-Thr_797_, and between the hydroxyl group at the C-1 and C-19 positions with l-Gln_111_ and C-11 and C-5 with l-Asn_122_ and l-Glu_117_, respectively. Thus, Ob was docked into NKA as expected, i.e., with its polar surface facing the polar amino acids. Similarly, the nonpolar surface of Ob was oriented towards the nonpolar part of the NKA cavity.

Another CG docked into NKA, the second reference ligand Dg, interacted like Ob with l-Thr_797_ via the β-hydroxyl group at the C-14. Unlike Ob, Dg contains only one of the aforementioned β-hydroxyl groups at the C-14, but even so, the orientation of its steroid skeleton was identical to that of Ob, underlining the importance of nonpolar interactions (especially with l-Phe_783_ and l-Leu_793_) for CG binding to NKA.

Similarly, the other CG ligands were docked to the binding site of NKA with the same steroid skeleton orientation as Ob and Dg; β-Hydroxyl groups at the C-14 position interacting with l-Thr_797_ were also present in the NKA-deHyr and NKA-Cy complexes. In the case of Cy, interactions of the carbonyl group at the C-19 and β-hydroxyl group at the C-5 with l-Gln_111_ and l-Glu_117_, respectively, were also found. The interaction of the conserved β-hydroxyl group of C-14 with l-Thr_797_ was also present in the NKA-deHyr complex. However, it was not present in the NKA-Hyr complex; on the contrary, this hydroxyl group interacted with l-Glu_117_, which lies closer to the NKA cavity surface. The absence of Hyr interaction with l-Thr_797_ of NKA was because Hyr did not penetrate deep enough into the CG binding site of NKA as the other evaluated ligands. This is probably caused by the presence of β-d-glucopyranosyl as a second saccharide unit (with the first being β-d-xylopyranosyl in the case of both Hyr and deHyr, as is illustrated in Figure 1) at the C-3 position of Hyr. Hyr interactions with the key amino acid residues (l-Glu_115_, l-Glu_116_, and l-Arg_886_) of NKA were detected only from the β-d-glucopyranosyl unit, not from the β-d-xylopyranosyl, as it was in the case of deHyr interaction with l-Glu_116_ and l-Glu_117_.

For other glycosylated ligands (Ob, Dg, Cy), the saccharide moiety also contributed to their binding to NKA. Ob and Cy interacted with l-Arg_880_ and l-Glu_312_ by α-l-rhamnopyranosyl present in Ob and β-d-cymaropyranosyl present in Cy. In the case of Dg, there was an interaction of the first β-d-digitoxopyranose with l-Arg_880_ and l-Asp_884_ of NKA and the third β-d-digitoxopyranose with l-Arg_886_.

To summarize, all ligands (Ob, Dg, deHyr, Hyr, and Cy) that docked into the NKA binding site differed in the type of glycosylation at the C-3 position, as well as in the number of hydroxyl and carbonyl groups present at the steroid skeleton. As is evident from our findings, the number of these groups is important for the strength of the bond with NKA. Ob, which contains the most hydroxyl groups in its structure and is, therefore, able to form the most hydrogen bridges with NKA, was docked with the lowest binding energy (Table 1). The importance of these interactions, especially with l-Gln_111_ and l-Asn_122_, is documented by the crystal structure of NKA with Ob in [45], and in several mutagenesis studies in which a significant reduction in CG affinity to NKA was observed due to their substitution.

Dg, deHyr, and Cy have also docked as Ob to the CG binding site in NKA with the same orientation of the steroid skeleton as Ob in the aforementioned NKA crystal structure [45]. The same orientation of the steroid skeleton was also observed in the crystal structure of NKA with digoxin [46] and strebloside, which is a structural analog of Cy [47]. However, deHyr and Cy showed higher binding energies compared to Ob caused by a lower number of interactions with NKA, which is due to the lower number of substituents on the steroid skeleton. For the same reason, the reference ligand Dg also had higher binding energy compared to Ob. As for Hyr, it was also docked with the same steroid skeleton orientation as Ob and Dg, however, its binding energy was higher not only compared to these ligands, but also compared to other glycosylated ligands, including deHyr, which contains only one saccharide unit in its structure. Precisely due to the presence of two saccharide units, Hyr did not dock as deeply into the NKA binding cavity as did the other tested ligands. It has been reported that the number of carbohydrate units affects the strength of the NKA–CG interaction [48], and, for this reason, there was an increase in binding energy for Hyr compared to deHyr. However, this argument does not apply to Dg, which contains trisaccharide in its structure, meaning that the size of the binding energy is determined by a combination of both factors, i.e., the appropriate type of substituents on the steroid skeleton and the degree of glycosylation. Overall, Hyr was docked with higher binding energy due to both the different number of substituents on the steroid skeleton and the different degrees of glycosylation compared to other glycosylated ligands.

### 3.3. Anticancer Potential of the Evaluated Steroid Glycosides

CGs as well-established therapeutics for the treatment of cardiac insufficiencies and arrhythmias have lately been subjected to drug repurposing, since it has been reported that they also exhibit great anticancer potential. Often, however, high systemic toxicity was also described. However, this phenomenon could be circumvented by higher cancer cell selectivity and/or a partial decrease in the overall toxicity. Therefore, as a next step, we evaluated the cytotoxicity and cancer cell selectivity of the CGs, Hyr, and deHyr (compared with Ob, Cy, and Dg), isolated from *C. varia* in a panel of human cancer cell lines. The activity was compared with toxicity results from noncancerous primary human cells, as well as mouse cells, which should be, in general, less sensitive to CGs due to different NKA isoforms.

The cytotoxicities of the CGs after 72 h of incubation were expressed as the half-maximal inhibitory concentrations (IC_50_), which are summarized in Table 2. The results showed marked differences in *in vitro* toxicity between the CGs both in potency and selectivity. For all compounds, we detected a concentration-dependent cytotoxicity profile in all evaluated cell lines. From the results, it is obvious that Hyr and deHyr, even though they exhibited lower cytotoxicities to cancerous cell lines than that of well-described CGs Ob and Dg, manifested a good selectivity for cancer cells when compared to noncancerous cells. As for Hyr, the most pronounced selectivity (compared against MRC-5 cells) was observed for human cancer cells from lung (A549), pancreas (MiaPaCa-2), breast (MCF-7), and transformed kidney cells (HEK 293T). Fairly good selectivity was also detected for human leukemic cells, cells from colorectal carcinoma regardless of *p53* deletion, and cells from bladder carcinoma. Concerning deHyr, it exhibited the highest cancer cell selectivity (compared to BJ cells) for A549 cells, which was followed by good selectivity for leukemic, pancreatic, breast, prostate, and colorectal (with and without *p53* deletion) cancer cells. Dg and Cy shared almost identical selectivity (compared to BJ cells) to cancer cells derived from lung and colon carcinoma, as well as to leukemic cells (Ob mainly to A549 cells).

As expected, cytotoxicity of the tested CGs was significantly affected by the type of attached saccharide at the C-3 position of the steroid skeleton. Based on the data gained in this study, we concluded that the derivatives with one attached carbohydrate moiety exhibited the highest cytotoxicity compared to derivatives glycosylated to a higher extent, which is in agreement with what is known for the sugar vs. cytotoxicity relationship for CGs in general [13]. However, interestingly, the least toxic CG, Hyr, contains two saccharide moieties. It exhibited even lower toxicity than Dg, which contains trisaccharide. This contradiction might be explained by the molecular docking into NKA, in which the presence of the third carbohydrate unit stabilized Dg in the NKA cavity to a greater extent than in the case of Hyr.

Besides the number of saccharide moieties, the distribution of substituents on the steroid skeleton of CGs also significantly affected their overall cytotoxicity. As aforementioned in the docking part of this study, the binding site for CGs is divided in terms of amino acid distribution into a polar and nonpolar part, which means that the presence of polar substituents on the steroid skeleton affects the level of NKA inhibition to a greater extent. Of the monoglycosylated CGs in this study, the highest cytotoxicity was exhibited by Cy, which, like deHyr, contains a carbonyl group at the C-19, but also contains a β-hydroxyl group at the C-5 position. In contrast, Ob contains β-hydroxyl group at the C-1, α-OH at C-10, and C-19 positions, but it lacks the β-OH at the C-5. Thus, it seems that the β-hydroxyl group of the C-5 might contribute to the overall cytotoxicity. However, as evidenced by Levrier et al., the cytotoxicity is also significantly affected by the carbonyl group at the C-19; when a hydroxyl group at the C-19 is substituted for a carbonyl moiety, the cytotoxicity of the resulting derivative increases approximately 150-fold [49]. The carbonyl group is also present in Cy, which was the most cytotoxic from the CGs in this study. Therefore, based on our data, we conclude that the cytotoxicity of the five CGs evaluated was mainly influenced by the carbonyl group at the C-19, the β-hydroxyl group at the C-5, and the decreasing number of carbohydrate units.

The trend of the dose response curves for the evaluated CGs was somewhat similar for all examined human cell lines. Nonetheless, the situation substantially differed for mouse fibroblasts (L929; see Table 2), for which none of the CGs exhibited any signs of cytotoxicity up to the highest tested concentration (10 µM), which is probably caused by increased resistance of the mouse α-subunit of NKA to CGs [50]. Amino acid substitutions Q111R and N122D are present in the murine α-subunit of NKA [51] and, therefore, mouse cells can tolerate up to at least 1000 times higher concentrations of CGs than corresponding human cells [52].

### 3.4. Steroid Glycoside Toxicity to Mouse Macrophages

**Figure 5:**
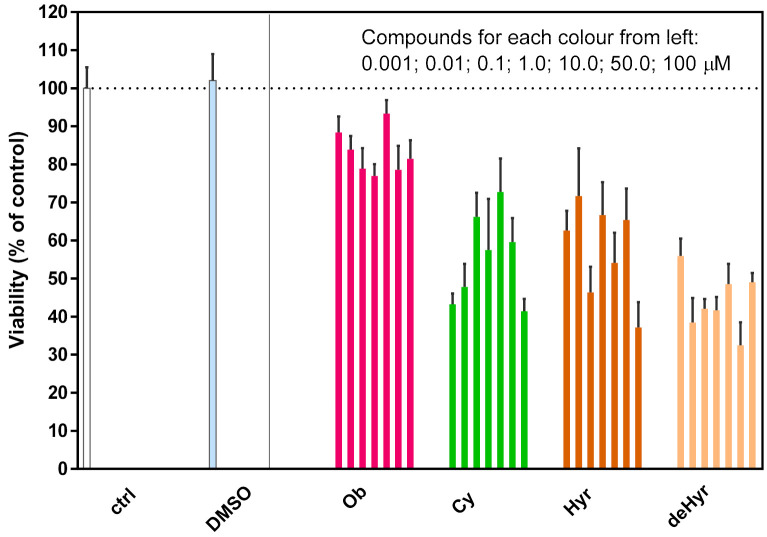
Viability of mouse peritoneal cells. Isolated cells were cultured for 24 h. Individual compounds, i.e., ouabain (Ob), cymarin (Cy), hyrcanoside (Hyr), and deglucohyrcanoside (deHyr), were applied at concentrations of 0.001, 0.01, 0.1, 1.0, 10.0, 50.0, and 100 µM. The effect of DMSO was also analyzed; its concentration corresponded to 50 µM concentration of compounds. WST-1 assay was used for viability evaluation. The results are expressed as the percent of untreated controls (ctrl) ± SEM (standard error of the mean) of n = 8 values from two independent experiments.

Even though it is known that CGs trigger immunogenic cell death and that they stimulate the immune response, not much is known about their effect on primary macrophages. For the first time, we bring data on the effect of Ob, Hyr, deHyr, and Cy on these cells: primary macrophages, the innate immune cells. For this task, the cells were isolated directly from mice, and cell viability after 24-h CG treatment was determined by WST-1. The results are summarized in Figure 5. It is obvious that the viability of mouse macrophages was reduced in the presence of all tested compounds in comparison to untreated control cells, but, for some, only marginally. The viability of mouse macrophages treated with Ob decreased only by 10–20% (compared to the control) quite independently on the used concentration (1 nm–100 µM), while cytotoxicity of Cy and Hyr was more pronounced—about 60% of the control. The most cytotoxic to mouse macrophages was deHyr, which reduced their viability to 50% of the control. Surprisingly, a dose-dependent decrease in cell viability was not found for any of the compounds, despite a wide range of tested concentrations: 0.001–100.0 µM.

To summarize, in our in vitro conditions, the most cytotoxic was deHyr; Cy and Hyr made a position between deHyr and Ob. Cytotoxicity of Ob was previously studied in neuronal-like SH-SY5Y cells after 24 and 48 h, for which Ob reduced their viability by 40 and 10%, respectively [53]. Distinct effects of Ob on the survival of human and rat vascular smooth muscle cells, endothelial cells, and astrocytes were confirmed by Akimova et al. [54]. Unlike human cells, their rodents counterparts perfectly survived in the presence of high concentrations of Ob (3–3000 μM), despite the complete inhibition of the NKA and inversion of the [Na^+^]_I_/[K^+^]_i_ ratio. These dramatic differences in Ob effects on rat and mouse cells are driven by variations of rodent α1 NKA isoform. This difference could also explain the non-existing dose-dependent curve in mouse macrophages.

It is generally accepted that CGs, such as clinically used digoxin, are cytotoxic. This is a rational reason to employ such compounds in anticancer therapy instead of the regulation of cardiac function. It seems that some cancer cell lines (especially the ones derived from lung and colon carcinoma) are more sensitive to cardenolides consisting of a lactone ring with five carbons than noncancerous cells. Recent data also document that another lesser known cardenolide derivative, nerigoside, was more cytotoxic in two colorectal cancer cell lines HT29 and SW620 when compared to normal human epithelial cell line NCM460 (determined by a similar in vitro viability assay, as in our study) [55]. Our pilot results showed that the cytotoxic effect of the tested CGs on mouse macrophages was not fully devastating, but the results significantly differed from mouse fibroblasts, for which no toxicity was detected up to 10 µM concentration, while for mouse macrophages, only Ob did not exhibit significant cytotoxicity (up to 100 µM concentration), which corresponds to the known fact that mouse cells are generally insensitive or less sensitive to CGs than human cells based on the expression of different NKA isoforms. Contrary to L929, in mouse macrophages, Cy reached IC_50_ already at the lowest tested concentration of 1 nM, deHyr ca. at 10 nM, and Hyr at ca. 100 nM concentration. The reason for this difference between the two types of mouse cells remains elusive.

### 3.5. Cell Cycle and Cell Death Analysis

Next, we wanted to find out whether Hyr and deHyr can arrest the cell cycle of cancer cells, as has been previously reported for Dg and Ob, which were evaluated in cancer cells of various origin. For a more detailed description of the biological activity of the studied derivatives, we performed cell cycle analysis of the most sensitive CCRF-CEM cells after 24 h of CG treatment (Table 3).

After 24-h incubation with 1× IC_50_ of CGs, CCRF-CEM cells were still viable. The percentage of the sub G0/1 population was only slightly increased compared to the untreated control, while treatment with 5× IC_50_ caused a typical increase in the cell number present in the sub G0/G1 phase, together with augmented DNA fragmentation. All five evaluated CGs induced an increase in the G2/M phase population. The decrease in cells in the S and G0/G1 populations was caused by the proportional accumulation of cells in the G2/M phase. We did not observe positivity for pH3^Ser10^, and negativity indicated G2 arrest. Such a finding is consistent with a recently reported study showing ATR-CHK2-CDC25C-mediated G2/M cell cycle arrest after Dg treatment [56].

BrDU is incorporated into newly synthesized DNA, and BrDU pulse labeling is therefore commonly used as a proliferation marker. Low BrDU incorporation into the DNA of treated cells with all compounds at 5× IC_50_ reflected inhibition of DNA synthesis, indicating irreversible apoptotic changes. The percentage of BrU positive cells incorporating 5-bromouridine is proportional to the transcriptional activity of CCRF-CEM cells. These values significantly decreased upon treatment with 5× IC_50_ of CGs, but not with 1× IC_50_.

Previously, the ability to arrest the cell cycle of cancer cells in the G2/M phase has been already described for Ob, oleandrin, digoxin, Dg, and its synthetic analog monoD [26,57,58,59]. In this study, the same effect was observed for all tested CGs, although in some, there was an increase in the G2/M phase already at 1× IC_50_, and in others only at 5× IC_50_. For substances in which the G2/M phase increased at 1× IC_50_, on the contrary, it decreased at 5× IC_50_, which means that each substance had its concentration optimum, above which G2/M decreased again. The same trend was observed by Elbaz et al. [26]. Lower incorporation of BrDU and BrU at 5× IC_50_ concentrations indicated that DNA and RNA synthesis decreased at these concentrations, although in some cases the number of S-phase cells may be higher, even though nucleic acid synthesis is no longer present and thus, the incorporation of BrDU and BrU decreases.

## 4. Conclusions

In this work, we describe a procedure for the isolation of two CGs, Hyr and deHyr, by aqueous EtOH extraction from seeds, with an overall yield of 3.3% and 0.0002%, respectively. This was an improvement by several times of magnitude than what has been reported so far. Both CGs, Hyr and deHyr, were assessed for their anticancer activity, which was compared to other well-known CGs Ob, Dg, and Cy. From the results, it is clear that Ob and deHyr outperformed the other CGs, which correlates with the docking study into NKA. The highest anticancer (based on the cancer cell selectivity) potential of all CGs was found against cells derived from lung and colorectal carcinoma. Moreover, all evaluated CGs arrested the cell cycle of CRF-CEM in the G2/M phase. Thus, even though further elaboration in deciphering detailed mechanisms of Hyr and deHyr and other CG anticancer activity is needed, the first results already indicate that these CGs could have a potential for a therapeutic application in cancer treatment. From the number of publications on CGs as anticancer drugs, it is obvious that they have come into the foreground as candidates of anticancer therapy with new mechanisms of actions than the standardly used chemotherapeutics, such as antimitotics [60] or cisplatin, and that their induction of the immune system response brings another added value in the treatment. We assume that our results can contribute to further development of drugs based on NKA interaction, and maybe additional molecular targets with selective cytotoxicity for cancer cells.

## Figures and Tables

**Table 1:** Binding energies of cardiac glycosides docked into Na^+^/K^+^-ATPase.

Ligand *	Binding Energies (kcal·mol^−1^)
Ob	−9.85
deHyr	−8.48
Dg	−7.47
Cy	−6.42
Hyr	−5.85

* Ob = ouabain; deHyr = deglucohyrcanoside; Dg = digitoxin; Cy = Cymarin; Hyr = Hyrcanoside.

**Table 2:** Summary of cytotoxic activities (IC_50_, nM) of the examined cardiac glycosides: ouabain (Ob), cymarin (Cy), digitoxin (Dg), hyrcanoside (Hyr), and deglucohyrcanoside (deHyr) after 72 h of incubation with cancerous and noncancerous human and mouse cells.

Compound	Ob	Cy	Dg	Hyr	deHyr
Cell Line	IC_50_ [nM] ^a^				
CCRF-CEM	32 ± 5.8	14 ± 2.1	20 ± 3.5	660 ± 38	110 ± 21
K562	57 ± 6.2	25 ± 4.5	19 ± 2.2	800 ± 52	130 ± 21
A549	22 ± 0.94	15 ± 1.8	19 ± 2.0	550 ± 38	90 ± 14
HCT116	30 ± 3.6	21 ± 2.5	20 ± 1.5	670 ± 37	140 ± 16
HCT116p53-/-	28 ± 7.6	19 ± 3.8	27 ± 9.6	730 ± 98	130 ± 23
MiaPaCa-2	49 ± 2.01	44 ± 8.6	79 ± 2.8	491 ± 23	120 ± 5.2
MCF-7	52 ± 5.6	29 ± 8.1	78 ± 1.1	566 ± 29	119 ± 6.4
U-2 OS	59 ± 1.1	43 ± 1.8	45 ± 2.1	1104 ± 59	165 ± 11
5637	110 ± 11	89 ± 5.5	58 ± 1.6	1511 ± 51	398 ± 46
PC-3	49 ± 1.4	26 ± 2.9	69 ± 2.2	756 ± 29	122 ± 4.7
HEK 293T	35 ± 1.2	3.02 ± 1.4	69 ± 2.1	383 ± 8.9	38 ± 0.97
MRC-5	39 ± 1.6	26 ± 2.4	78 ± 1.9	302 ± 43	77 ± 12
BJ	54 ± 9.9	54 ± 10	47 ± 12	1450 ± 250	230 ± 35
L929	>10,000	>10,000	>10,000	>10,000	>10,000

^a^ Cytotoxic activity was determined by MTS assay following 72 h of incubation. The values represent the mean of IC_50_ from three independent experiments. The tested cell lines: CCRF-CEM (childhood T-cell acute lymphoblastic leukemia), K562 (chronic myeloid leukemia), A549 (lung adenocarcinoma), HCT116 (colorectal carcinoma), HCT116p53-/- (HCT116 with deleted *p53* gene), MiaPaCa-2 (adenocarcinoma of pancreas), MCF-7 (breast carcinoma), U-2 OS (osteosarcoma), 5637 (bladder carcinoma), PC-3 (prostate carcinoma), and HEK 293T (transformed kidney cells). Noncancerous human cells: MRC-5 (lung fibroblasts) and BJ (fibroblasts from foreskin). L929, mouse transformed fibroblasts. The colors in the first slope represent the types of the cells: orange—human cancerous or transformed cell lines, blue—human primary noncancerous cells, green—mouse cells.

**Table 3:** Effect of cytotoxic compounds on cell cycle, apoptosis, and DNA/RNA synthesis in CCRF-CEM lymphoblasts (% of positive cells). Flow cytometry analysis was used to quantify cell cycle distribution and the percentage of apoptotic cells. The sum of the percentages for G0/G1, S, and G2/M is equal to 100%. ^a^ Phospho-Histone3 (Ser10); ^b^ BrDU, 5-bromo-2-deoxyuridine; ^c^ BrU, 5-bromouridine.

Compound *	< G1	G0/G1	S	G2/M	M ^a^	BrDU ^b^	BrU ^c^
Control	2.74	43.36	33.86	22.34	1.69	33.10	34.51
Ob 1 × IC_50_	20.68	40.42	25.78	33.80	1.73	40.53	39.77
Ob 5 × IC_50_	33.31	33.94	40.80	25.27	0.63	19.29	6.56
Cy 1 × IC_50_	2.89	35.78	39.87	24.35	1.43	32.34	35.63
Cy 5 × IC_50_	7.01	40.34	31.17	28.49	1.08	21.37	27.52
Dg 1 × IC_50_	7.56	33.21	36.28	30.51	1.97	35.91	42.99
Dg 5 × IC_50_	10.35	33.71	22.26	44.03	1.28	23.52	3.88
Hyr 1 × IC_50_	2.82	38.48	35.22	26.30	1.41	30.58	28.80
Hyr 5 × IC_50_	5.82	42.13	23.01	34.86	0.95	23.58	3.82
deHyr 1 × IC_50_	22.02	44.90	17.60	37.50	1.44	37.40	38.91
deHyr 5 × IC_50_	27.43	39.12	34.53	26.34	1.56	6.08	12.17

* Ob = ouabain; deHyr = deglucohyrcanoside; Dg = digitoxin; Cy = Cymarin; Hyr = Hyrcanoside.

## Supplementary Materials

The following are available online at https://www.mdpi.com/2304-8158/10/1/136/s1; Figure S1: Other known components of *C. varia*; Figure S2: Structures related to naming mismatch; Figure S3: Structures of so-called “securigenin glycosides”; Figure S4: The ^1^H and ^13^C chemical shifts of deHyr in CD_3_OD and CD_3_SOCD_3_; Figure S5: The ^1^H and ^13^C chemical shifts of Hyr in CD_3_OD and CD_3_SOCD_3_; Figures S6 and S7: MS spectrum of Hyr and deHyr, respectively; Figures S8 and S9: HPLC chromatograms of Hyr and deHyr, respectively; Table S1: ^1^H and ^13^C NMR characteristics of Hyr (securigenin s3), deHyr and related securigenins s4 and s5.
