# Supplementary material for: Steroid Glycosides Hyrcanoside and Deglucohyrcanoside: On Isolation, Structural Identification, and Anticancer Activity
Source: Foods. 2021 Jan 11;10(1):136. doi: 10.3390/foods10010136 (PMC7827417; doi:10.3390/foods10010136)
Supplement: Supplementary file 1 [file foods-10-00136-s001.pdf]

Supporting information

Steroid glycosides hyrcanoside and deglucohyrcanoside: On isolation, structural identification and anticancer activity

Silvie Rimpelová [1ł], Tomáš Zimmermann [2ł], Pavel B. Drašar [2], Bohumil Dolenský [3], Jiří Bejček [1], Eva Kmoníčková [5,6], Petra Cihlářová [2], Soňa Gurská [4], Lucie Kuklíková [1], Marián Hajdůch [4], Tomáš Ruml [1], Lubomír Opletal [7], Petr Džubák [4*] and Michal Jurášek [2*]

[1] Department Biochemistry and Microbiology, University of Chemistry and Technology Prague, Technická 5, 166 28 Prague 6, The Czech Republic; silvie.rimpelova@vscht.cz; jiri.bejcek@vscht.cz; lucie.kuklikova@vscht.cz; tomas.ruml@vscht.cz

[2] Department of Chemistry of Natural Compounds, University of Chemistry and Technology Prague, Technická 5, 166 28 Prague 6, The Czech Republic; tomas.zimmermann@vscht.cz, michal.jurasek@vscht.cz, pavel.drasar@vscht.cz

[3] Department of Analytical Chemistry, University of Chemistry and Technology Prague, Technická 5, 166 28 Prague 6, The Czech Republic; bohumil.dolensky@vscht.cz

[4] Institute of Molecular and Translational Medicine, Faculty of Medicine and Dentistry, Palacký University Olomouc, Hněvotínská 976/3, 779 00 Olomouc, The Czech Republic; petr.dzubak@upol.cz, sona.gurska@upol.cz, marian.hajduch@upol.cz

[5] Department of Pharmacology and Toxicology, Faculty of Medicine in Pilsen, Charles University, Alej Svobody 76, 323 00 Pilsen, Czech Republic; eva.kmonickova@lfp.cuni.cz

[6] Department of Pharmacology, Second Faculty of Medicine, Charles University, Plzeňská 311, 150 00 Prague, Czech Republic; eva.kmonickova@lfmotol.cuni.cz

[7] Department of Pharmaceutical Botany, Charles University, Akademika Heyrovskeho 1203, 500 05 Hradec Králové, The Czech Republic; opletal@faf.cuni.cz

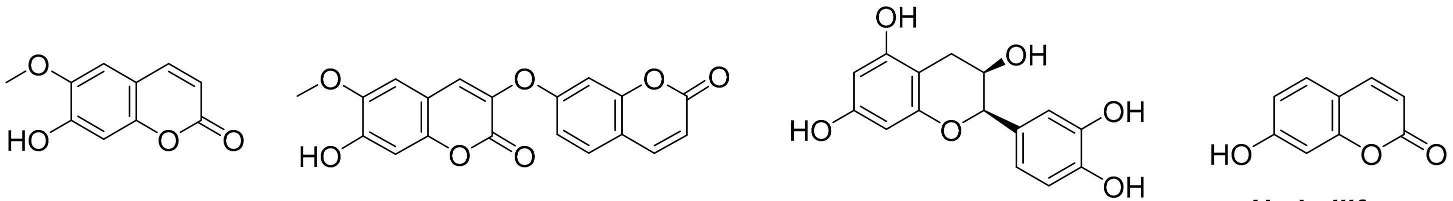

Scopoletin **Daphnoretin** **(-)-Epicatechin** **Umbelliferone**

Figure S1. Other known components of *C. varia* [1]

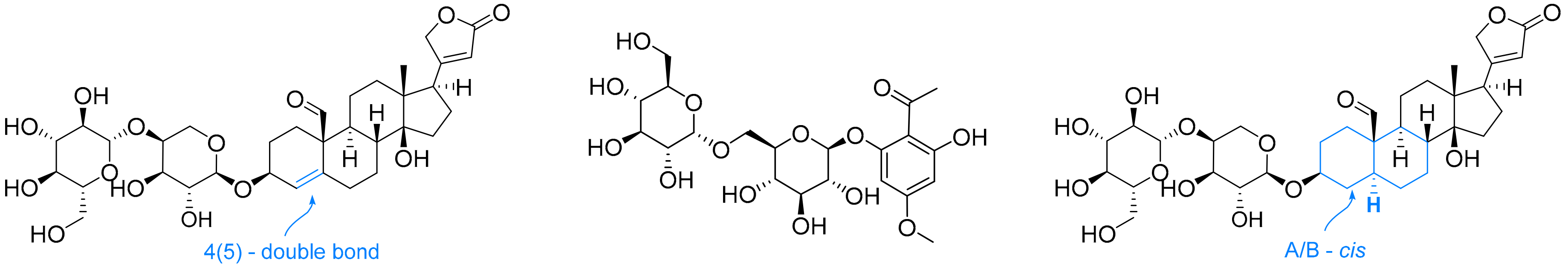

Hyr **s1** **s2**

Figure S2. Structures related to different nomenclature (s1 [2, 3]; s2 [4-6])

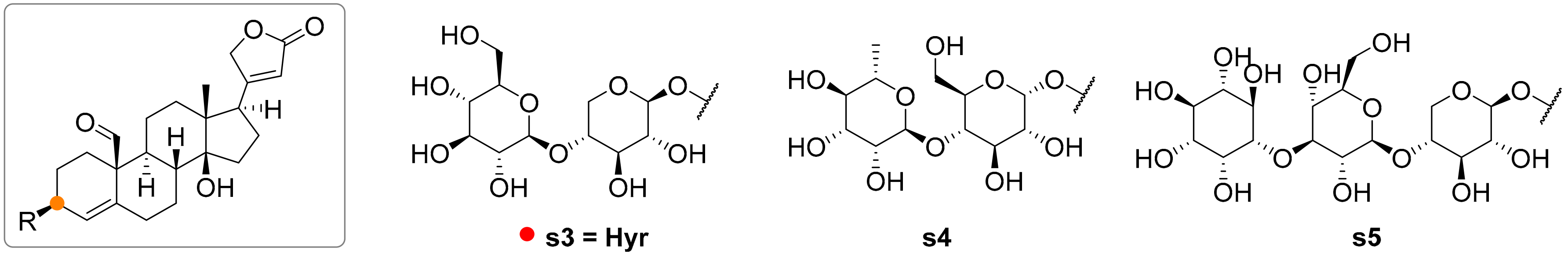

● **s3 = Hyr** **s4** **s5**

Figure S3. Structures of so-called „securigenin glycosides“ [7]

Table S1. ^{1}H and ^{13}C NMR characteristics of **Hyr** (securigenin **s3**), **deHyr**, and related securigenins **s4** and **s5** (Figure S3)

	**Hyr**<br>**in CD_3OD**	**s3** [7] **(Hyr)**<br>**in CD_3OD**	**s4** [7]<br>**in CD_3OD**	**s5** [7]<br>**in CD_3OD**	**deHyr**<br>**in CD_3OD**	**deHyr**<br>**in DMSO-*d*$_6$**	**deHyr** [8]<br>**in DMSO-*d*$_6$**	**Hyr**<br>**in DMSO-*d*$_6$**	**Hyr** [8]<br>**in DMSO-*d*$_6$**
C1	29.695	29.7	29.6	29.6	29.707	27.981	27.92	27.713	27.61
H1a	1.366 m covered	1.30 m	1.35 m	1.32 m	1.356 m	1.253 m		1.258 m	
H1b	2.433 m covered	2.38 m	2.44 m	2.40 m	2.432 m	2.318 m		2.323 m	
C2	28.695	28.7	28.7	28.4	28.695	27.432	27.50	27.163	27.46
H2a	2.023 m	1.99 m	2.04 m	2.01 m	2.022 m	1.875 m		1.882 m	
H2b	1.423 m	1.34 m	1.39 m	1.37 m	1.428 m	1.199 m		1.194 m	
C3	75.845	75.8	75.8	76.0	75.835	73.079	73.16	72.875	73.32
H3	4.198 ddt<br>$^{3}J_{H3,H2b}$ = 9.4<br>$^{3}J_{H3,H2a}$ = 5.8<br>$^{3,5}J_{H3,H6b+H4}$ = 2.1	4.16 t<br>7.4	4.21 t<br>7.4	3.21 dd<br>8.9<br>8.0	4.192 dddd<br>$^{3}J_{H3,H2b}$ = 9.5<br>$^{3}J_{H3,H2a}$ = 5.7<br>$^{3}J_{H3,H4}$ = 2.4<br>$^{5}J_{H3,H6b}$ = 1.7	4.076 dddd<br>$^{3}J_{H3,H2b}$ = 9.5<br>$^{3}J_{H3,H2a}$ = 5.8<br>$^{3}J_{H3,H4}$ = 2.4<br>$^{5}J_{H3,H6b}$ = 1.6		4.090 ddt<br>$^{3}J_{H3,H2b}$ = 9.6<br>$^{3}J_{H3,H2a}$ = 5.8<br>$^{4,5}J_{H3,H4+H6b}$ = 2.0	
C4	127.740<br>$^{1}J_{C4,H4}$ ~ 156	127.7	127.7	127.3	127.775<br>$^{1}J_{C4,H4}$ ~ 157	126.573	126.62	126.258	126.59
H4	5.819 dt<br>$^{4}J_{H4,H6b}$ ~ 2.1<br>$^{3,4}J_{H4,H2a+H3}$ ~ 1.3	5.78 s	5.83 s	5.70 s	5.818 ~q<br>$^{3,4}J_{H4,H2a+H3+H6b}$ ~1.7	5.699 ~q<br>$^{3,4}J_{H4,H2a+H3+H6b}$ ~1.6		5.699 br q<br>$^{3,4}J_{H4,H2a+H3+H6b}$<br>~1.5	
C5	140.042	140.0	140.0	140.1	140.013	137.936	137.98	137.772	138.10
C6	34.153	34.1	34.1	34.1	34.154	32.546	28.05	32.303	27.92
H6a	2.317 ddd<br>$^{2}J_{H6a,H6b}$ = 14.2<br>$^{3}J_{H6a,H7a}$ = 4.3<br>$^{3}J_{H6a,H7b}$ = 2.9	2.28 m	2.34 m	2.30 m	2.317 ~ddd<br>$^{2}J_{H6a,H6b}$ = 14.1<br>$^{3}J_{H6a,H7a}$ = 4.3<br>$^{3}J_{H6a,H7b}$ = 2.9	2.226 ~ddd<br>$^{2}J_{H6a,H6b}$ = 14.1<br>$^{3}J_{H6a,H7a}$ = 4.3<br>$^{3}J_{H6a,H7b}$ = 2.8		2.226 ~ddd<br>$^{2}J_{H6a,H6b}$ ~ 14.1<br>$^{3}J_{H6a,H7a}$ ~ 4.3<br>$^{3}J_{H6a,H7b}$ ~ 2.9	
H6b	2.392 tdt<br>$^{2,3}J_{H6b,H6a+H7a}$ ~ 13.6<br>$^{3}J_{H6b,H7b}$ = 4.6<br>$^{4,5}J_{H6b,H4+H3}$ ~ 2.1	2.33 m	2.39 m	2.35 m	2.391 tdt covered<br>$^{2,3}J_{H6b,H6a+H7a}$ ~ 13.7<br>$^{3}J_{H6b,H7b}$ = 4.6<br>$^{4,5}J_{H6b,H4+H3}$ ~ 2.1	2.303 br t covered<br>$^{2,3}J_{H6b,H6a+H7a}$ ~ 13.7		2.307 tdt<br>covered<br>$^{2,3}J_{H6b,H6a+H7a}$ ~<br>13.2<br>$^{3}J_{H6b,H7b}$ = 4.5<br>$^{4,5}J_{H6b,H4+H3}$ ~ 2.0	
C7	29.366	29.3	29.3	29.3	29.370	27.754	26.36	27.511	26.39
H7a	1.139 dtd<br>$^{2}J_{H7a,H6b}$ = 13.5<br>$^{2,3}J_{H7a,H7b+H8}$ ~ 12.4<br>$^{3}J_{H7a,H6a}$ = 4.3	1.10 m	1.15 m	1.13 m	1.140 dtd<br>$^{2}J_{H7a,H6b}$ = 13.4<br>$^{2,3}J_{H7a,H7b+H8}$ ~ 12.4<br>$^{3}J_{H7a,H6a}$ = 4.3	0.987 dtd<br>$^{2}J_{H7a,H6b}$ = 13.4<br>$^{2,3}J_{H7a,H7b+H8}$ ~ 12.2<br>$^{3}J_{H7a,H6a}$ = 4.3		0.987 dtd<br>$^{2}J_{H7a,H6b}$ = 13.5<br>$^{2,3}J_{H7a,H7b+H8}$ ~<br>12.6	

								$^{3}J_{H7a,H6a}$ = 4.3	
H7b	2.201 ddt<br>$^{2}J_{H7b,H7a}$ = 12.6<br>$^{3}J_{H7b,H6b}$ = 4.6<br>$^{3}J_{H7b,H6a+H8}$ = 2.9	2.15 m	2.21 m	2.19 m	2.201 ddt<br>$^{2}J_{H7b,H7a}$ = 12.6<br>$^{3}J_{H7b,H6b}$ = 4.6<br>$^{3}J_{H7b,H6a+H8}$ = 2.9	2.150 ddt<br>$^{2}J_{H7b,H7a}$ = 12.6<br>$^{3}J_{H7b,H6b}$ = 4.7<br>$^{3}J_{H7b,H6a+H8}$ = 2.9		2.152 ddt<br>$^{2}J_{H7b,H7a}$ = 12.5<br>$^{3}J_{H7b,H6b}$ = 4.3<br>$^{3}J_{H7b,H6a+H8}$ = 2.9	
C8	43.985	43.9	43.9	43.9	43.996	42.293	31.52	42.049	31.59
H8	1.889 td<br>$^{3}J_{H8,H7a+H9}$ = 12.0<br>$^{3}J_{H8,H7b}$ = 3.1	1.84 m	1.89 m	1.86 m	1.887 td<br>$^{3}J_{H8,H7a+H9}$ = 12.1<br>$^{3}J_{H8,H7b}$ = 2.9	1.682 td<br>$^{3}J_{H8,H7a+H9}$ ~ 11.8<br>$^{3}J_{H8,H7b}$ = 2.9		1.683 td<br>$^{3}J_{H8,H7a+H9}$ ~ 11.7<br>$^{3}J_{H8,H7b}$ = 3.0	
C9	50.806	50.8	50.8	50.7	50.814	48.808	49.86	48.563	49.89
H9	1.326 ~tm covered<br>$^{3}J_{H9,H8+H11b}$ ~ 11.8	1.27 m	1.32 m	1.29 m	1.325 ~tm covered<br>$^{3}J_{H9,H8+H11b}$ ~ 11.6	1.249 m covered		1.245 m<br>covered	
C10	54.848	54.8	54.8	54.8	54.854	53.208	53.25	52.970	53.25
C11	22.759	22.7	22.7	22.7	22.761	21.226	21.32	20.980	21.29
H11a	1.695 m	1.65 m	1.70 m	1.68 m	1.705 m	1.582 m		1.583 m	
H11b	~ 1.45 m covered	1.39 m	1.43 m	1.41 m	~ 1.45 m	1.277 m		1.276 m<br>covered	
C12	40.561	40.5	40.5	40.5	40.566	38.578	32.64	38.339	31.68
H12a	~ 1.44 m covered	1.36 m	1.41 m	1.38 m	~ 1.44 m	1.302 m		1.299 m<br>covered	
H12b	1.539 ~dd<br>$^{3}J_{H12b,H12a}$ ~ 9.9<br>$^{3}J_{H12b,H11a}$ ~ 3.2	1.49 m	1.54 m	1.52 m	1.539 ~dd<br>$^{3}J_{H12b,H12a}$ ~ 9.5<br>$^{3}J_{H12b,H11a}$ ~ 3.0	1.423 ~dd<br>$^{3}J_{H12b,H12a}$ ~ 9.8<br>$^{3}J_{H12b,H11a}$ ~ 2.7		1.426 m ~dd<br>$^{3}J_{H12b,H12a}$ ~ 9.7<br>$^{3}J_{H12b,H11a}$ ~ 2.6	
C13	50.798	50.8	50.8	50.7	50.806	49.117	49.17	48.874	49.18
C14	85.676	85.6	85.6	85.7	85.685	83.198	83.28	82.953	83.29
OH	-				-	4.274 br s		4.274 br<br>covered	
C15	32.701	32.7	32.7	32.7	32.703	31.453	28.05	31.208	28.05
H15a	1.627 ddd<br>$^{2}J_{H15a,H15b}$ = 13.4<br>$^{3}J_{H15a,H16b}$ = 9.0<br>$^{3}J_{H15a,H16a}$ = 1.7	1.58 m	1.64 m	1.61 m	1.628 ddd<br>$^{2}J_{H15a,H15b}$ = 13.4<br>$^{3}J_{H15a,H16b}$ = 9.1<br>$^{3}J_{H15a,H16a}$ = 1.8	1.507 ddd<br>$^{2}J_{H15a,H15b}$ = 13.0<br>$^{3}J_{H15a,H16b}$ = 8.8<br>$^{3}J_{H15a,H16a}$ = 1.8		1.507 ddd<br>$^{2}J_{H15a,H15b}$ = 12.8<br>$^{3}J_{H15a,H16b}$ = 8.8<br>$^{3}J_{H15a,H16a}$ = 1.8	
H15b	2.032 dt<br>$^{2}J_{H15b,H15a}$ = 13.4<br>$^{3}J_{H15b,H16a+H16b}$ = 9.9	1.99 m	2.04 m	2.01 m	2.032 dt<br>$^{2}J_{H15b,H15a}$ = 13.4<br>$^{3}J_{H15b,H16a+H16b}$ = 9.9	1.887 dt<br>$^{2}J_{H15b,H15a}$ = 13.0<br>$^{3}J_{H15b,H16a+H16b}$ = 9.7		1.886 covered<br>$^{2}J_{H15b,H15a}$ = 13.3<br>$^{3}J_{H15b,H16a+H16b}$ =<br>9.7	
C16	27.911	27.9	27.9	27.9	27.915	26.268	27.81	26.030	27.92

H16a	2.150 dtd<br>$^{2}J_{H16a,H16b}$ = 13.3<br>$^{3}J_{H16a,H15b+H17}$ = 9.8<br>$^{3}J_{H16a,H15a}$ = 1.7	2.09 m	2.15 m	2.13 m	2.150 dtd<br>$^{2}J_{H16a,H16b}$ = 13.4<br>$^{3}J_{H16a,H15b+H17}$ = 9.9<br>$^{3}J_{H16a,H15a}$ = 1.8	1.998 dtd<br>$^{2}J_{H16a,H16b}$ = 13.2<br>$^{3}J_{H16a,H15b+H17}$ = 9.7<br>$^{3}J_{H16a,H15a}$ = 1.7		1.998 dtd<br>$^{2}J_{H16a,H16b}$ = 13.0<br>$^{3}J_{H16a,H15b+H17}$ =<br>9.7<br>$^{3}J_{H16a,H15a}$ = 1.7	
H16b	1.870 dtd<br>$^{2}J_{H16b,H16a}$ = 13.3<br>$^{3}J_{H16b,H15b+H15a}$ = 9.3<br>$^{3}J_{H16b,H17}$ = 5.5	1.84 m	1.89 m	1.86 m	1.870 dtd<br>$^{2}J_{H16b,H16a}$ = 13.4<br>$^{3}J_{H16b,H15b+H15a}$ = 9.0<br>$^{3}J_{H16b,H17}$ = 5.5	1.779 dtd<br>$^{2}J_{H16b,H16a}$ = 13.2<br>$^{3}J_{H16b,H15b+H15a}$ = 9.2<br>$^{3}J_{H16b,H17}$ = 5.3		1.775 dtd<br>$^{2}J_{H16b,H16a}$ = 13.1<br>$^{3}J_{H16b,H15b+H15a}$ =<br>9.3<br>$^{3}J_{H16b,H17}$ = 5.3	
C17	51.748	51.7	51.7	51.6	51.756	49.785	48.90	49.537	48.91
H17	2.825 dd<br>weak COSY to<br>H22<br>$^{3}J_{H17,H16a}$ = 9.6<br>$^{3}J_{H17,H16b}$ = 5.5	2.78 dd<br>9.5<br>5.6	2.84 dd<br>9.5<br>5.6	2.81 dd<br>9.5<br>5.6	2.826 dd<br>weak COSY to<br>H22 and H21a<br>$^{3}J_{H17,H16a}$ = 9.6<br>$^{3}J_{H17,H16b}$ = 5.5	2.726 dd<br>weak COSY to<br>H22 and H21a<br>$^{3}J_{H17,H16a}$ = 9.5<br>$^{3}J_{H17,H16b}$ = 5.3		2.726 dd<br>$^{3}J_{H17,H16a}$ = 9.5<br>$^{3}J_{H17,H16b}$ = 5.4	
C18	16.203	16.2	16.2	16.2	16.203	15.487	15.54	15.424	15.55
H18	0.892 s<br>weak COSY to<br>H17 and H12a	0.85 s	0.90 s	0.85 s	0.892 s	0.764 s		0.764 s	
C19	205.381	205.4	205.5	205.5	205.392	204.815	204.79	204.568	204.82
H19	9.817 s<br>weak COSY to H9<br>$^{1}J_{H19,C19}$ = 173.5<br>$^{2}J_{H19,C10}$ = 20.5	9.78 s	9.83 s	9.83 s	9.816 d<br>$^{4}J_{H19,H9}$ = 0.8<br>$^{1}J_{H19,C19}$ = 173.6<br>$^{2}J_{H19,C10}$ = 20.5	9.798 s<br>weak COSY to<br>H9<br>$^{1}J_{H19,C19}$ = 174.4<br>$^{2}J_{H19,C10}$ = 20.7		9.800 s<br>weak COSY to<br>H9<br>$^{1}J_{H19,C19}$ = 174.5<br>$^{2}J_{H19,C10}$ = 20.7	
C20	178.172	178.2	178.2	178.2	178.180	176.118	176.17	175.877	176.17
C21	75.319	75.3	75.3	75.3	75.324	73.122	73.23	72.881	73.16
H21a	5.020 ddd<br>$^{2}J_{H21a,H21b}$ = 18.4<br>$^{4}J_{H21a,H22}$ = 1.9<br>$^{4}J_{H21a,H17}$ = 0.7	4.98 dd<br>18.4<br>1.6	5.04 dd<br>18.4<br>1.6	5.02 dd<br>18.4<br>1.6	5.024 ddd<br>$^{2}J_{H21a,H21b}$ = 18.4<br>$^{4}J_{H21a,H22}$ = 1.8<br>$^{4}J_{H21a,H17}$ = 0.7	4.944 ddd [a]<br>$^{2}J_{H21a,H21b}$ = 18.4<br>$^{4}J_{H21a,H22}$ = 1.9<br>$^{4}J_{H21a,H17}$ = 0.7		4.942 ddd<br>$^{2}J_{H21a,H21b}$ = 18.4<br>$^{4}J_{H21a,H22}$ = 1.8<br>$^{4}J_{H21a,H17}$ = 0.7	
H21b	4.912 dd<br>$^{2}J_{H21b,H21a}$ = 18.4 Hz<br>$^{4}J_{H21b,H22}$ = 1.8 Hz	4.87 dd<br>18.4<br>1.6	4.93 dd<br>18.4<br>1.6	4.91 dd<br>18.4<br>1.6	4.912 dd<br>$^{2}J_{H21b,H21a}$ = 18.4 Hz<br>$^{4}J_{H21b,H22}$ = 1.8 Hz	4.882 dd [a]<br>$^{2}J_{H21b,H21a}$ = 18.4<br>$^{4}J_{H21b,H22}$ = 1.8		4.844 dd<br>$^{2}J_{H21b,H21a}$ = 18.4<br>$^{4}J_{H21b,H22}$ = 1.8	
C22	117.936<br>$^{1}J_{C22,H22}$ = 179.7	117.9	117.9	117.9	117.937<br>$^{1}J_{C22,H22}$ = 179.6	116.343	116.41	116.097	116.48
H22	5.902 td	5.87 s	5.93 s	5.90 s	5.902 td	5.912 td		5.912 td	

	$^{4}J_{H22,H21a+H21b}$ = 1.8<br>$^{4}J_{H22,H17}$ = 0.7				$^{4}J_{H22,H21a+H21b}$ = 1.8<br>$^{4}J_{H22,H17}$ = 0.7	$^{4}J_{H22,H21a+H21b}$ = 1.8<br>$^{4}J_{H22,H17}$ = 0.7		$^{4}J_{H22,H21a+H21b}$ =<br>1.8<br>$^{4}J_{H22,H17}$ = 0.7	
C23	177.207	177.2	177.2	177.3	177.217	173.824	173.85	173.584	173.87
C1'	104.211<br>$^{1}J_{C1',H1'}$ ~ 157	104.1	104.1	100.8	104.433<br>$^{1}J_{C1',H1'}$ = 155.8	102.797		102.259	
H1'	4.374 d<br>$^{3}J_{H1',H2'}$ = 7.6	4.34 d<br>7.9	4.40 d<br>7.9	4.88 d<br>1.5	4.349 d<br>$^{3}J_{H1',H2'}$ = 7.6	4.221 d<br>$^{3}J_{H1',H2'}$ = 7.7		4.273 d<br>$^{3}J_{H1',H2'}$ = 7.6	
C2'	74.804	74.7	74.7	72.4	74.948	73.264		72.875	
H2'	3.179 dd<br>$^{3}J_{H2',H3'}$ = 9.2<br>$^{3}J_{H2',H1'}$ = 7.6	3.14 m	3.19 m	3.82 dd<br>3.1<br>1.6	3.123 dd<br>$^{3}J_{H2',H3'}$ = 9.1<br>$^{3}J_{H2',H1'}$ = 7.6	2.885 ddd<br>$^{3}J_{H2',H3'}$ = 8.8<br>$^{3}J_{H2',H1'}$ = 7.7<br>$^{3}J_{H2',OH}$ = 4.5		2.952 br td<br>$^{3}J_{H2',H1'+H3'}$ = 8.3<br>$^{3}J_{H2',OH}$ = 3.6	
OH	-	-	-	-	-	4.968 br covered		5.111 br d<br>$^{3}J_{OH,H2'}$ = 5.1	
C3'	76.137	76.0	76.0	74.0	77.905	76.674		74.489	
H3'	3.461 t<br>$^{3}J_{H3',H2'+H4'}$ = 9.0	3.43 t<br>9.0	3.49 t<br>9.0	4.14 m	3.298 dd<br>$^{3}J_{H3',H2'}$ = 9.1<br>$^{3}J_{H3',H4'}$ = 8.8	3.075 td<br>$^{3}J_{H3',H2'}$ = 8.8<br>$^{3}J_{H3',OH}$ = 3.7		3.249 br td<br>$^{3}J_{H3',H2'+H4'}$ = 8.9<br>$^{3}J_{H3',OH}$ = 2.5	
OH	-	-	-	-	-	4.906 br covered		4.935 br<br>covered	
C4'	78.609	78.5	78.5	83.4	71.200	69.559		76.274	
H4'	3.665 ddd<br>$^{3}J_{H4',H5b'}$ = 10.2<br>$^{3}J_{H4',H3'}$ = 8.8<br>$^{3}J_{H4',H5a'}$ = 5.3	3.63 m	3.70 m	3.63 t<br>9.4	3.466 ddd<br>$^{3}J_{H4',H5b'}$ = 10.3<br>$^{3}J_{H4',H3'}$ = 8.8<br>$^{3}J_{H4',H5a'}$ = 5.3	3.247 ddd<br>$^{3}J_{H4',H5b'}$ = 10.3<br>$^{3}J_{H4',H3'}$ = 8.8<br>$^{3}J_{H4',H5a'}$ = 5.3<br>$^{3}J_{H4',OH}$ = 4.8		3.500 ddd<br>$^{3}J_{H4',H5b'}$ = 10.1<br>$^{3}J_{H4',H3'}$ = 8.8<br>$^{3}J_{H4',H5a'}$ = 5.3	
OH	-	-	-	-	-	4.914 br covered		-	
C5'	64.557	64.5	64.5	71.4	66.926	65.628		63.008	
H5a'	3.297 dd<br>$^{2}J_{H5b',H5a'}$ = 11.8<br>$^{3}J_{H5b',H4'}$ = 10.1	3.96 dd<br>11.7<br>5.3	4.02 dd<br>11.9<br>2.0	3.31 m	3.189 dd<br>$^{2}J_{H5b',H5a'}$ = 11.4<br>$^{3}J_{H5b',H4'}$ = 10.3	3.020 dd<br>$^{2}J_{H5b',H5a'}$ = 11.2<br>$^{3}J_{H5b',H4'}$ = 10.3		3.148 dd<br>$^{2}J_{H5b',H5a'}$ = 11.2<br>$^{3}J_{H5b',H4'}$ = 10.1	
H5b'	4.000 dd<br>$^{2}J_{H5a',H5b'}$ = 11.7<br>$^{3}J_{H5a',H4'}$ = 5.4	3.26 m	3.31 m	-	3.824 dd<br>$^{2}J_{H5a',H5b'}$ = 11.4<br>$^{3}J_{H5a',H4'}$ = 5.3	3.648 dd<br>$^{2}J_{H5a',H5b'}$ = 11.2<br>$^{3}J_{H5a',H4'}$ = 5.3		3.849 dd<br>$^{2}J_{H5a',H5b'}$ = 11.5<br>$^{3}J_{H5a',H4'}$ = 5.3	
C1''	103.468<br>$^{1}J_{C1',H1'}$ ~ 159	103.4	103.4	105.6				101.395	

H1''	4.355 d<br>$^{3}J_{H1'',H2''}$ = 7.8	4.32 d<br>8.0	4.38 d<br>8.0	4.59 br s	4.251 d<br>$^{3}J_{H1'',H2''}$ = 7.9
C2''	74.659	74.6	74.6	77.9	72.455
H2''	3.200 dd<br>$^{3}J_{H2'',H3''}$ = 9.2<br>$^{3}J_{H2'',H1''}$ = 7.8	3.16 m	3.21 m	3.31 m	3.000 br td<br>$^{3}J_{H2'',H1''+H3''}$ ~ 8.0<br>$^{3}J_{H2'',OH}$ ~ 2.4
OH	-	-	-	-	4.949 br<br>covered
C3''	77.851	77.7	77.7	78.0	76.129
H3''	3.350 t<br>$^{3}J_{H3'',H2''+H4''}$ = 8.8	3.34 m	3.39 m	3.40 m	3.138 br t<br>$^{3}J_{H3'',H2''+H4''}$ ~ 8.5
OH	-	-	-	-	4.950 br<br>covered
C4''	71.530	71.4	71.4	72.4	69.893
H4''	3.271 dd<br>$^{3}J_{H4'',H5''}$ = 9.7<br>$^{3}J_{H4'',H3''}$ = 8.4	3.24 m	3.29 m	3.86 m	3.023 br<br>covered
OH	-	-	-	-	4.928 br<br>covered
C5''	78.110	78.0	78.0	68.7	76.771
H5''	3.310 ~ddd<br>covered<br>$^{3}J_{H5'',H6a''}$ = ~ 9.7<br>$^{3}J_{H5'',H6b''}$ = ~ 5.7<br>$^{3}J_{H5'',H4''}$ = ~ 2.1	3.28 m	3.33 m	3.70 m	3.145 ddd<br>covered<br>$^{3}J_{H5'',H4''}$ ~ 9.8<br>$^{3}J_{H5'',H6b''}$ ~ 6.4<br>$^{3}J_{H5'',H6a''}$ ~ 2.2
C6''	62.624	62.6	62.6	-	60.887
H6a''	3.869 dd<br>$^{2}J_{H6a'',H6b''}$ = 11.9<br>$^{3}J_{H6a'',H5''}$ ~ 2.2	3.83 dd<br>11.9<br>2.0	3.89 dd<br>11.9<br>2.0	-	3.681 br dt<br>$^{2}J_{H6a'',H6b''}$ ~ 11.5<br>$^{3}J_{H6a'',H5''+OH}$ ~ 2.4
H6b''	3.644 dd<br>$^{2}J_{H6b'',H6a''}$ = 11.9<br>$^{3}J_{H6b'',H5''}$ = 5.9	3.60 m	3.66 m	-	3.403 br ddd<br>$^{2}J_{H6b'',H6a''}$ ~ 11.5<br>$^{3}J_{H6b'',H5''}$ ~ 6.3<br>$^{3}J_{H6b'',OH}$ ~ 2.3
OH	-	-	-	-	4.529 br t<br>$^{3}J_{OH,H6a''+H6b''}$ ~<br>2.4

[a] The signals H21a and H21b have almost identical NOE, therefore, they were assigned based on the similarity of the chemical shifts and multiplicity.

deHyr in CD_3OD

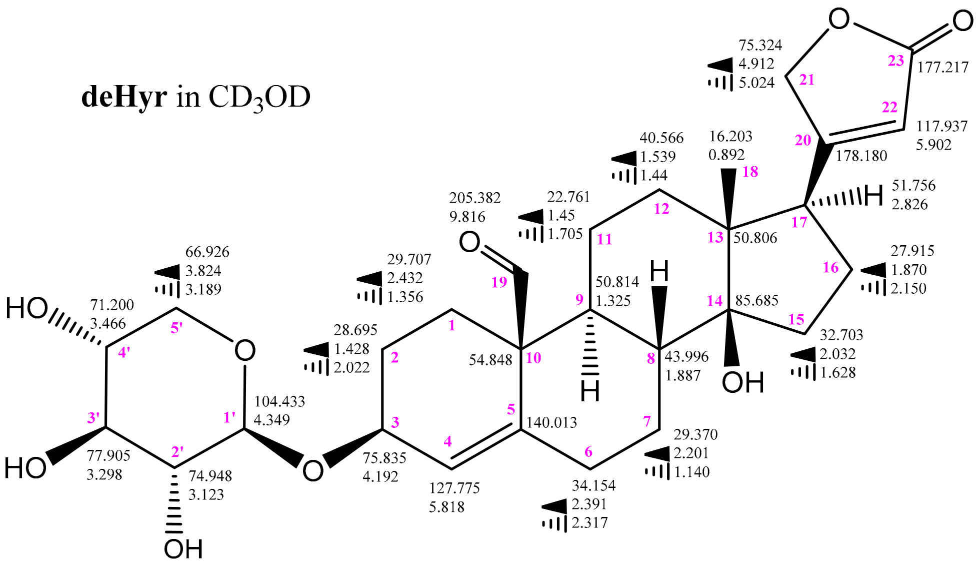

deHyr in DMSO-*d6*

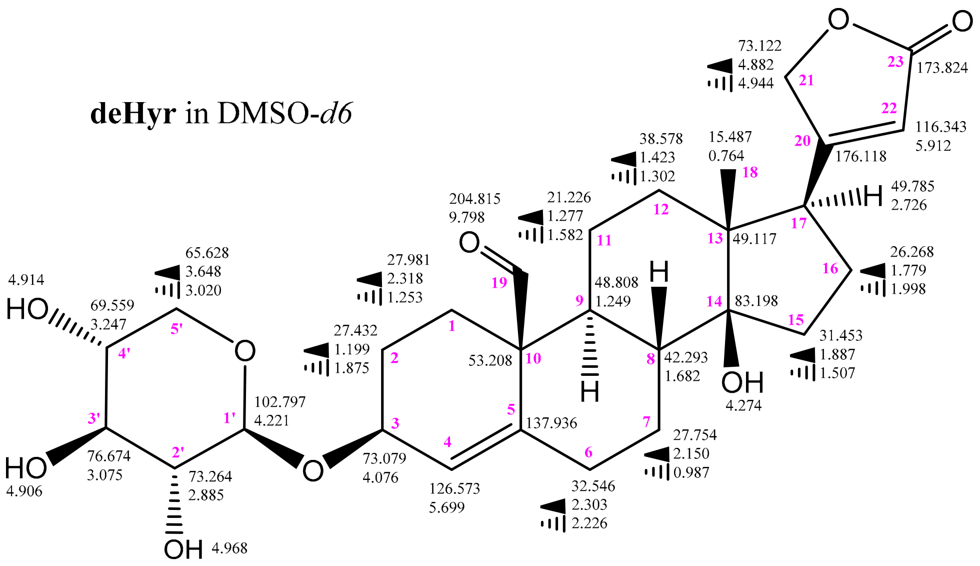

Figure S4. The ^{1}H and ^{13}C chemical shifts of **deHyr** in CD_3OD and DMSO-*d6*

Hyr in CD_3OD

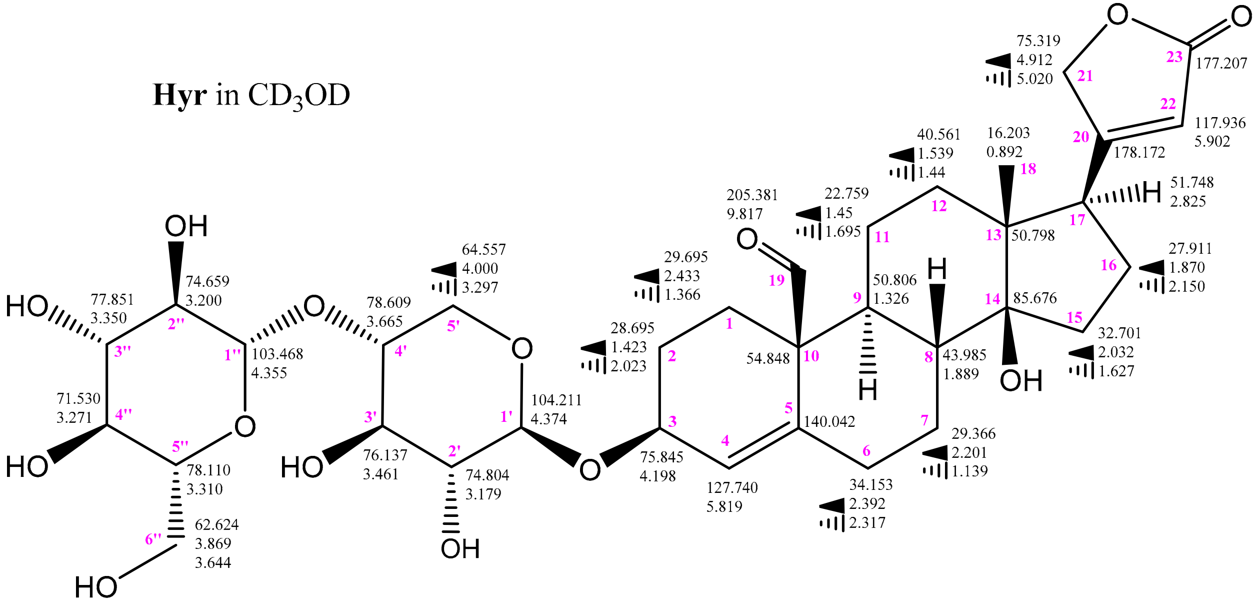

Hyr in DMSO-*d6*

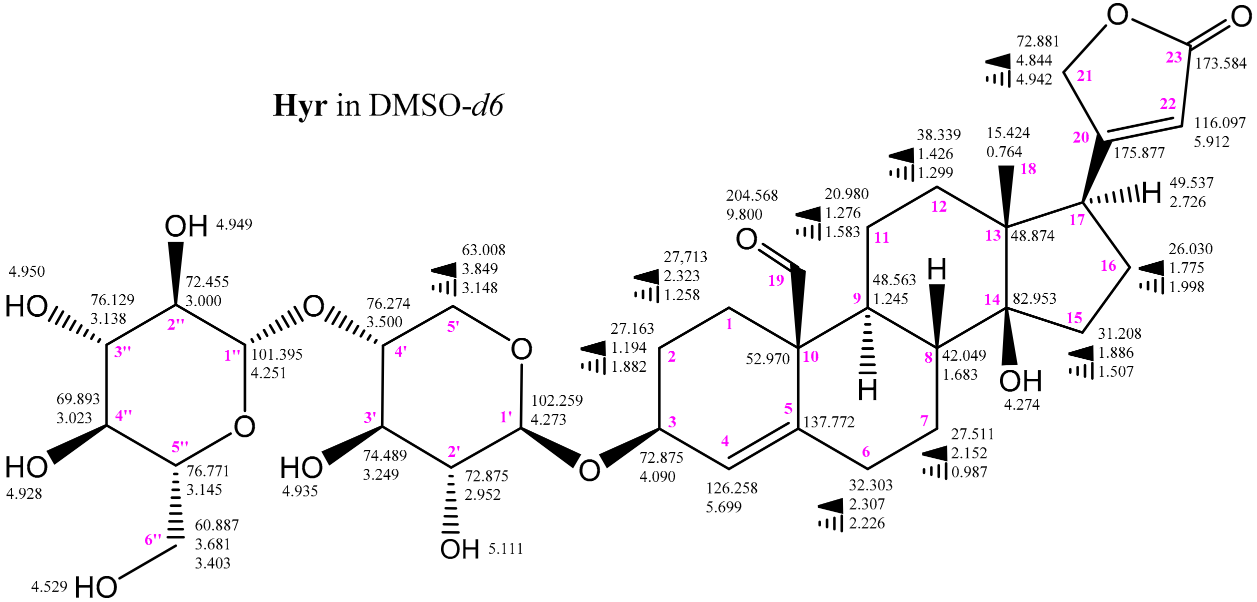

Figure S5. The ^{1}H and ^{13}C chemical shifts of **Hyr** in CD_3OD and DMSO-*d6*

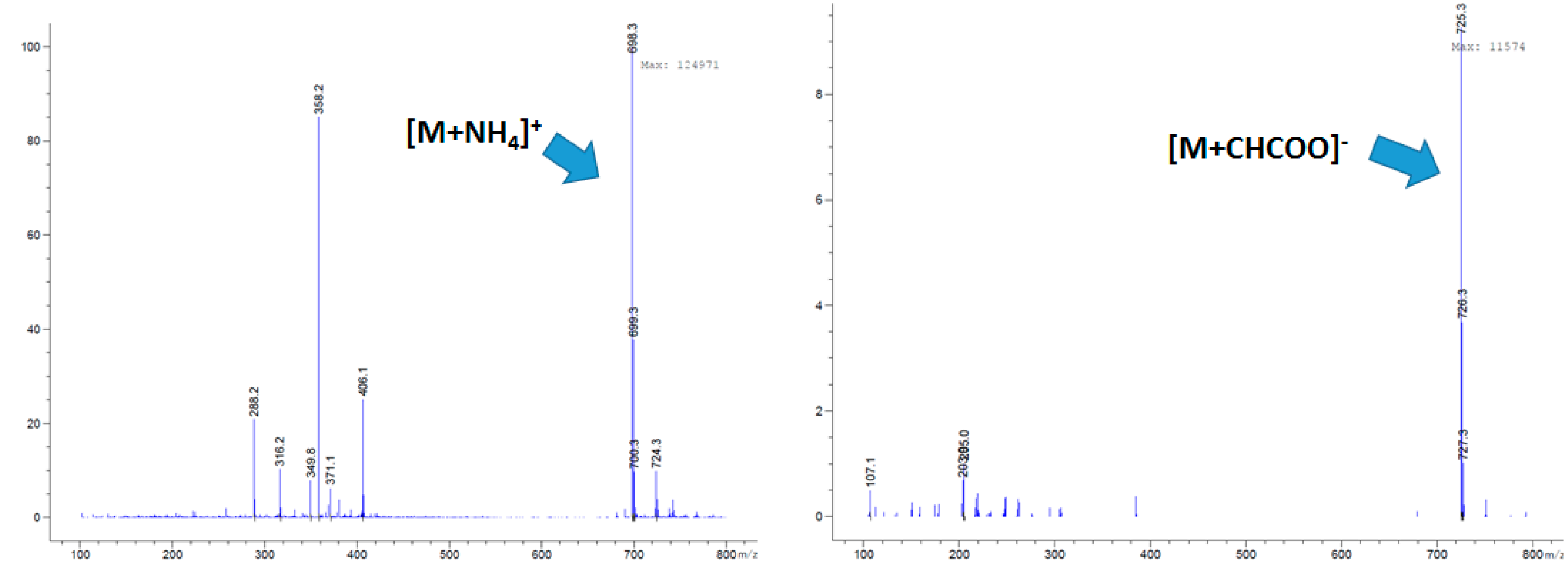

Figure S6. MS spectrum of **Hyr**

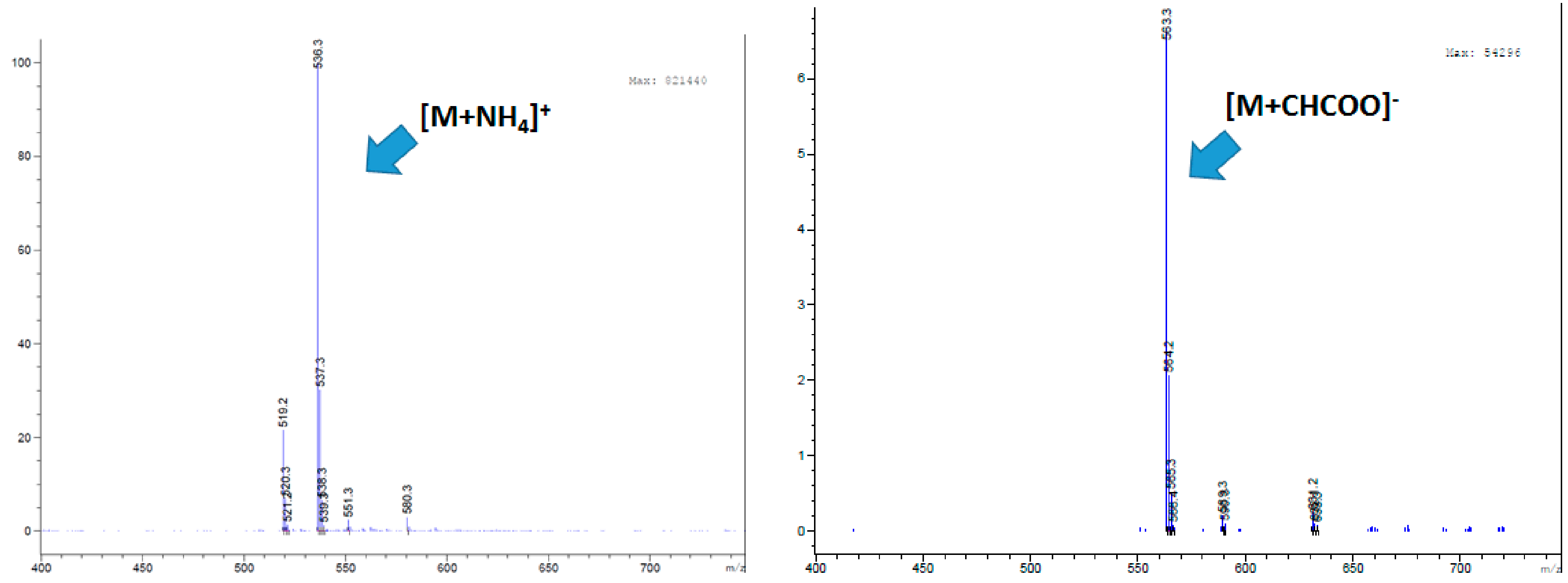

Figure S7. MS spectrum of **deHyr**

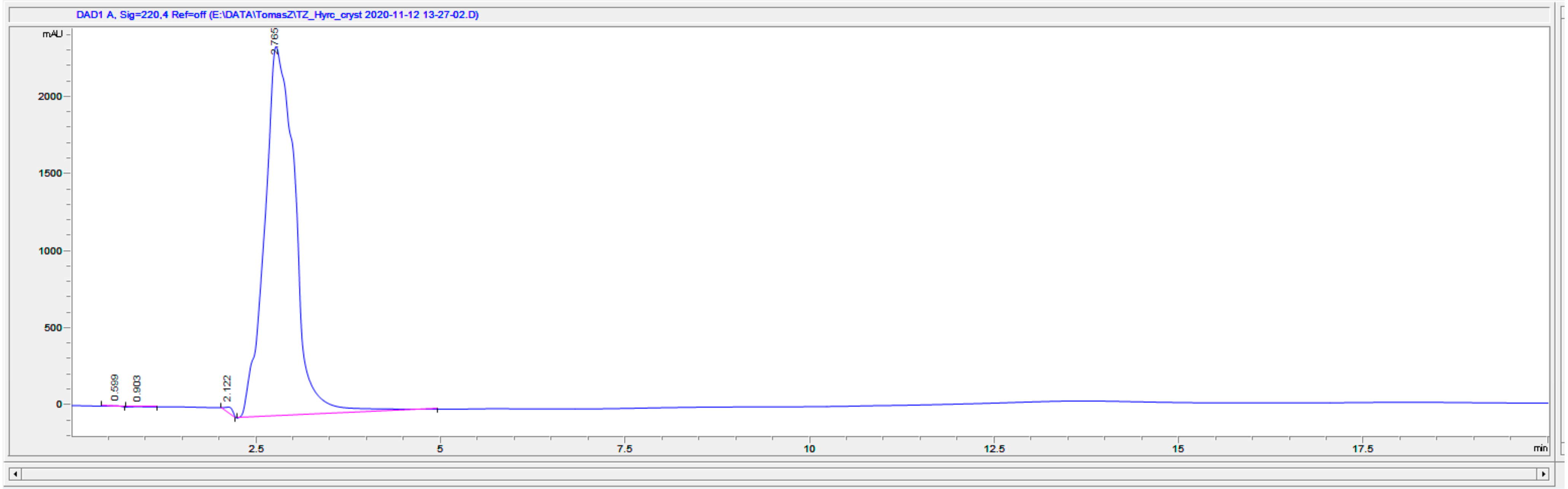

File Information

LC-File	TZ_Hyrc_cryst 2020-11-12 13-27-02.D
File Path	E:\DATA\TomasZ
Date	12-Nov-20, 13:27:59
Sample	TZ_Hyrc_cryst
Sample Info	
Barcode	
Operator	TOmas Zimmerman
Method	Screen-H2O-to-MeOH-UV-Kamil.M
Reference	
Analysis Time	20.007 min
Sampling Rate	0.0067 min (0.402 sec), 3002 datapoints

#	Time	Type	Area	Height	Width	Area%	Symmetry
1	0.599	BB	53.6	5.3	0.1661	0.077	1.204
2	0.903	BB	26.3	1.7	0.2294	0.038	0.927
3	2.122	BB	255.2	34.5	0.1144	0.367	0.701
4	2.765	BB	69276	2394.8	0.3735	99.519	0.607

Figure S8. HPLC chromatogram of **Hyr**

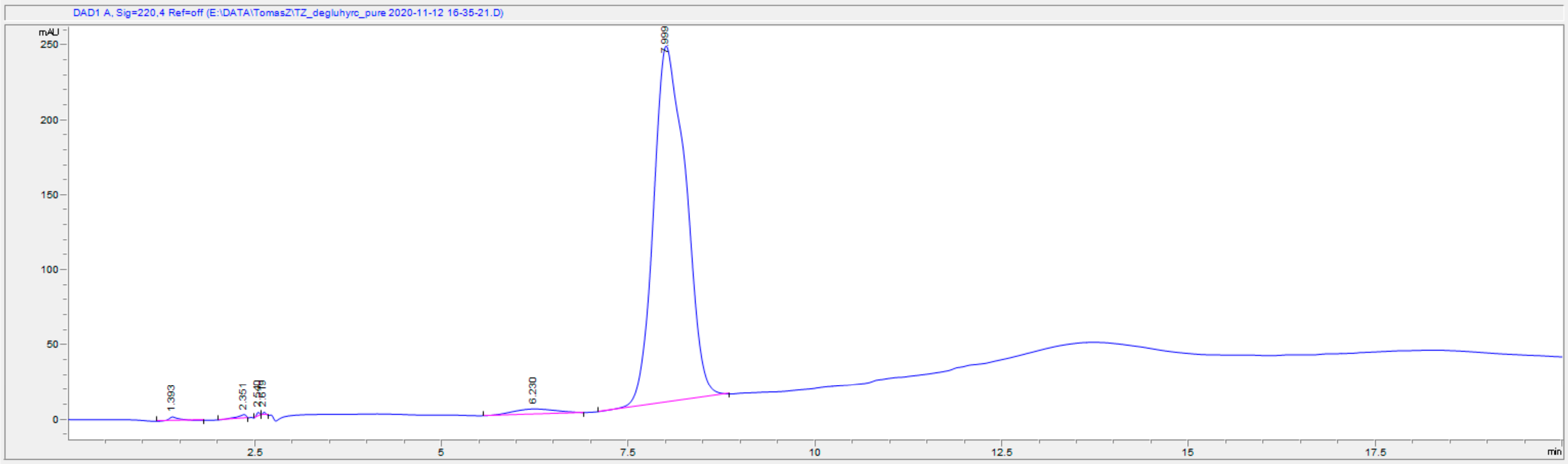

File Information

LC-File	TZ_degluhyrc_pure 2020-11-12 16-35-21.D
File Path	E:\DATA\TomasZ
Date	12-Nov-20, 16:36:09
Sample	TZ_degluhyrc_pure
Sample Info	
Barcode	
Operator	TOmas Zimmerman
Method	Screen-H2O-to-MeOH-UV-Kamil.M
Reference	
Analysis Time	20.007 min
Sampling Rate	0.0067 min (0.402 sec), 3002 datapoints

#	Time	Type	Area	Height	Width	Area%	Symmetry
1	1.393	BB	29	2.7	0.1495	0.395	0.494
2	2.351	BB	21	2.3	0.1266	0.286	3.959
3	2.54	BB	7	2.5	0.0474	0.096	1.451
4	2.619	BB	5.1	1.8	0.0468	0.069	0.746
5	6.23	BB	134.3	3.4	0.5328	1.831	0.954
6	7.999	BB	7139.8	238.2	0.4237	97.322	0.645

Figure S9. HPLC chromatogram of **deHyr**

References

[1] M. Williams, J. M. Cassady, Potential antitumor agents: A cytotoxic cardenolide from *Coronilla varia* L. *J. Pharm. Sci.* **1975**, 65, 912-914.

[2] M. R. Nurmukhamedova, G. K. Nikonov, Glycosides from *Dorema hyrcanum*. *Prirodnykh Soedinenii* **1976**, 3, 101-2.

[3] Khushbaktova, Z. A.; Mukhtasimova, R.; Syrov, V. N.; Sultanov, M. B., O farmakologicheskikh svoistvach novogo fenolglykozida – girkanozida [Pharmacological properties of a new phenolglycoside – hyrcanoside], *Doklady Akademii Nauk UzSSR* **1983**, 54-5.

[4] V. V. Zatula, N. P. Maksyutina, D. G. Kolesnikov, Cardenolides of *Securigera securidaca*. *Khimiya Prirodnykh Soedinenii* **1965**, 1, 153-156.

[5] V. V. Zatula, N. V. Chernobrovaya, D. G. Kolesnikov, A chemical study of the structure of securigenin and its bioside securidaside. *Khimiya Prirodnykh Soedinenii* **1966**, 2, 438-439.

[6] Zatula V. V., Kil'kisne vyznachennia sekurydazydu v nasinni sekuryhery mechovydnoi [Quantitative determination of securidaside in seeds of *Securigera securidaca*]. *Farmatsevtychnyi zhurnal* (Kiev) **1968**, 23, 85-88.

[7] Z. Tofighi, F. Moradi-Afrapoli, S. N. Ebrahimi, S. Goodarzi, A. Hadjiakhoondi, M. Neuburger, M. Hamburger, M. Abdollahi, N. Yassa, Securigenin glycosides as hypoglycemic principles of *Securigera securidaca* seeds, *J. Nat. Med.* **2017**, 71 (1), 272-280.

Note: *the stereo positions of hydrogen atoms were not estimated in the original article, they are suggested based on similarity of the chemical shifts to our determined values; the signals C3′ and H3′ are probably accidentally interchanged with the signals C5′′ and H5′′, resp., in the original article at least for* **Suc 1** (**Hyr**) *and* **Suc**.

[8] J. A. Hembree, C.-J. Chang, J. L. McLaughlin, G. Peck, J. M. Cassady, Potential antitumor agents: a cytotoxic cardenolide from *Coronilla varia*, *J. Nat. Prod.* **1979**, 42 (3), 293-298.

Note: *the signals C4 and H22 are probably interchanged in the original article*
